# Extracellular membrane vesicles in the three domains of life and beyond

**DOI:** 10.1093/femsre/fuy042

**Published:** 2018-11-21

**Authors:** Sukhvinder Gill, Ryan Catchpole, Patrick Forterre

**Affiliations:** 1 Institute for Integrative Biology of the Cell (I2BC), Biologie Cellulaire des Archées (BCA), CEA, CNRS, Université Paris-Sud, 91405 Orsay cedex, France; 2 Institut Pasteur, Unité de Biologie Moléculaire du Gène chez les Extrêmophiles, Département de Microbiologie, F75015 Paris, France

**Keywords:** extracellular vesicles, nanotubes, Archaea, virus, evolution, LUCA

## Abstract

Cells from all three domains of life, Archaea, Bacteria and Eukarya, produce extracellular vesicles (EVs) which are sometimes associated with filamentous structures known as nanopods or nanotubes. The mechanisms of EV biogenesis in the three domains remain poorly understood, although studies in Bacteria and Eukarya indicate that the regulation of lipid composition plays a major role in initiating membrane curvature. EVs are increasingly recognized as important mediators of intercellular communication via transfer of a wide variety of molecular cargoes. They have been implicated in many aspects of cell physiology such as stress response, intercellular competition, lateral gene transfer (via RNA or DNA), pathogenicity and detoxification. Their role in various human pathologies and aging has aroused much interest in recent years. EVs can be used as decoys against viral attack but virus-infected cells also produce EVs that boost viral infection. Here, we review current knowledge on EVs in the three domains of life and their interactions with the viral world.

## Teaser

This review discusses the current research on extracellular vesicle (EV) biology and their various roles in the three domains of life: Archaea, Bacteria and Eukarya. The physiological and/or evolutionary relationships between EVs and viruses are also examined.

## INTRODUCTION

**Figure 1.**
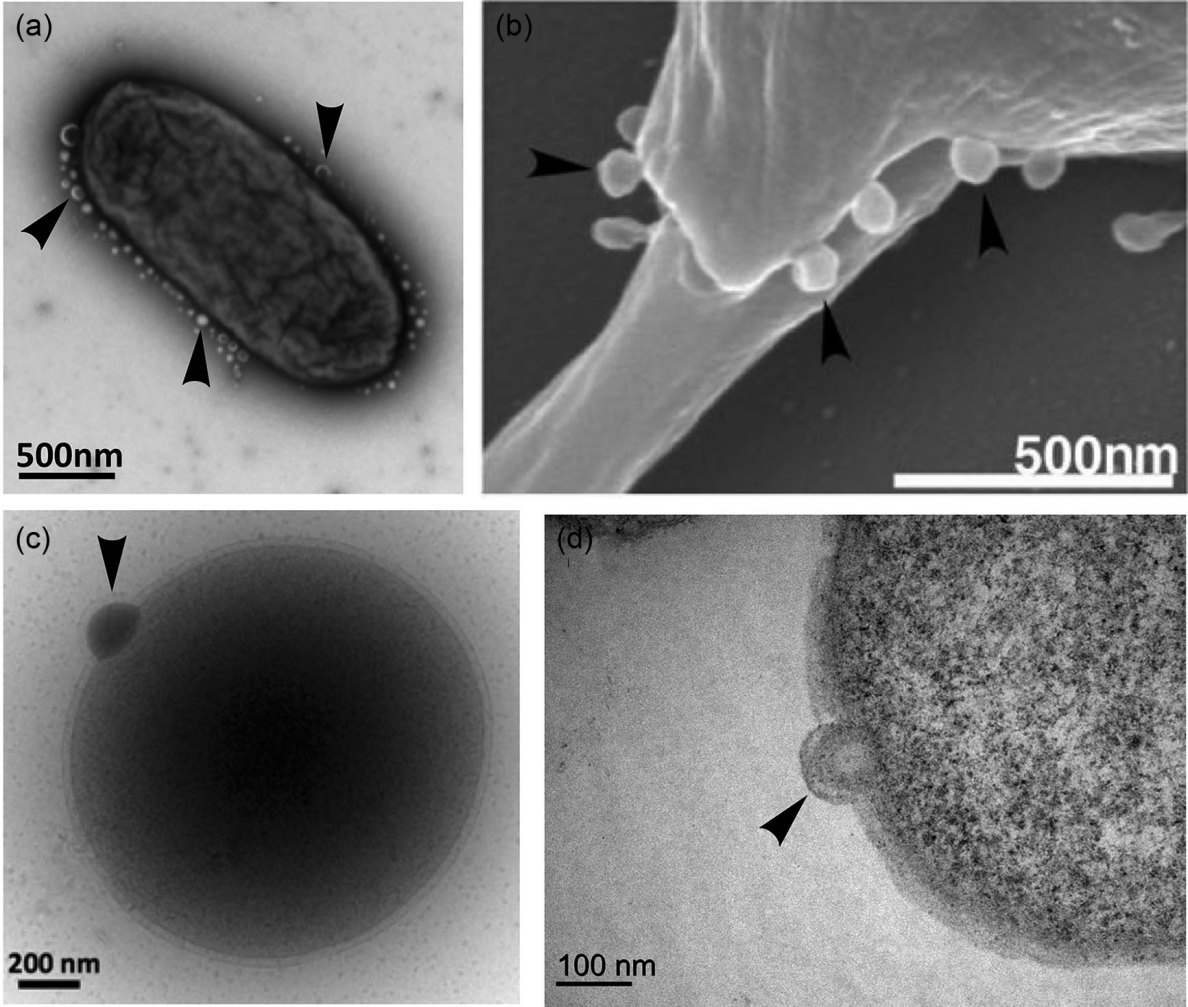
Biogenesis of extracellular vesicles in the three domains of life. Vesicle budding indicated with arrows. (**a**) TEM showing hypervesiculation in the bacterium *S. typhimurium*. Image kindly provided by Mario F. Feldman (University of Alberta, Canada). (**b**) SEM showing microvesicles budding from the eukaryote *Leishmania donovani.* Image reprinted from Silverman *et al.* (2008). (**c**) Cryo-TEM of vesicle budding from the archaeon *T. kodakaerensis.* The protrusion of the S layer can also be observed clearly. (**d**) TEM of ultrathin cell sections of vesicle budding from *T. kodakaerensis*. Figures (c) and (d) provided by the authors.

The release of membrane-bound vesicles is a universally conserved cellular process that occurs in all three domains of life (Deatherage and Cookson 2012; Schatz and Vardi 2018) (Fig. 1). The production of these extracellular vesicles (EVs) has been systematically observed each time researchers have investigated this phenomenon, suggesting that all cells are potentially capable of EV production. These EVs can transport various molecular cargoes and deliver them to recipient cells by fusion with the cytoplasmic membrane and/or by endocytosis in eukaryotes, modifying their physiology.

**Figure 2.**
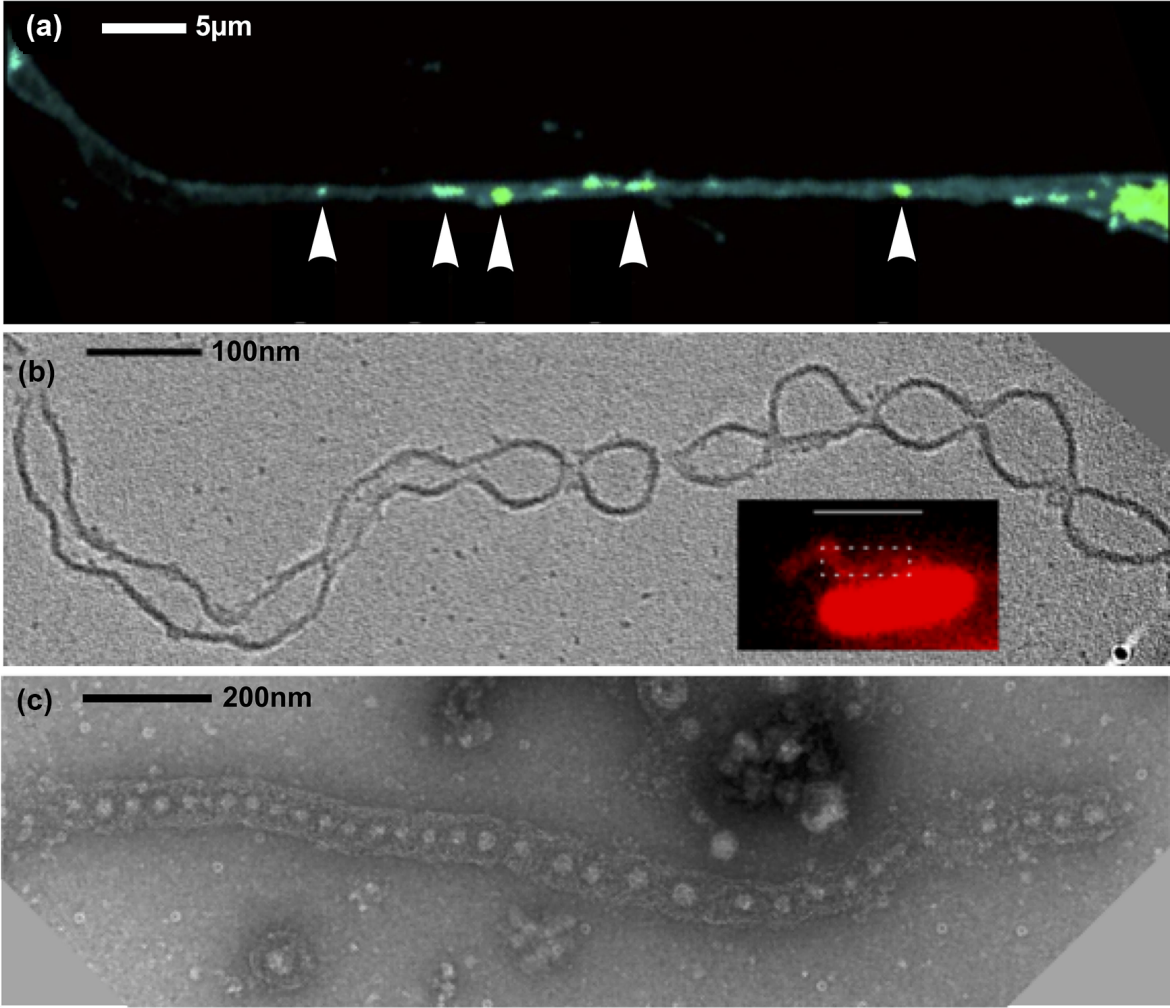
Nanotube production in the three domains of life. (**a**) TNT connecting eukaryotic (human) cells, with labeled vesicles indicated by arrows. Adapted with permission from Keller *et al.* (2017): image cropped and arrow style altered. (**b**) 'Nanotubes' produced by the bacteria *S. oneidensis* form outer membrane extensions with regular constrictions forming vesicles. Adapted with permission from Subramanian *et al.* (2018). Image courtesy of Poorna Subramanian (California Institute of Technology, USA). (**c**) 'Nanopods' produced by the archaeon *T. prieurii.* Discrete vesicles are surrounded by the cellular S-layer forming a tubular structure. Image kindly provided by Aurore Gorlas (Institute for Integrative Biology of the Cell, Université Paris-Saclay, France).

Because of their small size (from 20 to 500 nm for most of them), EVs have been mainly observed by electron microscopy, either as free particles in the culture medium following concentration or as nascent particles budding from the cell membranes (refer to Fig. 1). EVs observed by transmission electron microscopy (TEM) are usually heterogeneous in size with irregular shapes, such as a cup-shaped appearance, possibly due to sample preparation. However, they appear perfectly spherical when observed by cryo-electron microscopy without chemical fixation or contrasting (Koning *et al.*^2013^; Raposo and Stoorvogel 2013; Gorlas *et al.*^2015^; Pérez-Cruz *et al.*^2015^; Milasan *et al.*^2016^) (Fig. 1c). EVs are sometimes associated with long filamentous structures connecting cells in the three domains of life, known as nanopods and nanotubes in Archaea and Bacteria and tunneling nanotubes (TNTs) or ‘microvillus-like protrusions’ in Eukaryotes (Fig. 2) (Marguet *et al.*^2013^; Koistinen *et al.*^2015^; Keller *et al.*^2017^; Nawaz and Fatima 2017; Stempler *et al.*^2017^). These nanotubes often contain arrays of EVs surrounded by membranes, suggesting that they could be involved in EV production (Soler *et al.*^2008^; Dubey and Ben-Yehuda 2011; Shetty *et al.*^2011^; Marguet *et al.*^2013^). Notably, nanotubes seem to be involved in the production of EVs by some cancerous human cells (Rilla *et al.*^2013^).

The importance of EV production as a major phenomenon in the living world was for a long time underestimated, with EVs being initially dismissed as platelets or cellular ‘dust’ (Wolf 1967; Cocucci, Racchetti and Meldolesi 2009) and ignored in most microbiology textbooks. However, EV-focused research over the past two decades has begun to reveal their significance in cell physiology and their diverse biological functions have been extensively documented. It is now well recognized that EVs and related nanotubes can transport a variety of cargoes, including proteins, lipids, sugars and nucleic acids, and play important roles in all types of cell-to-cell interactions. The concentration of cargoes within membrane-bound EVs offers protection against extracellular enzymes and the aqueous environment and allows the secretion of both lipophilic and hydrophobic compounds. In particular, EVs are the only secretion system, proposed to be named secretion system type zero (Guerrero-Mandujano *et al.*^2017^), allowing cells to secrete and share with other cells lipids, hydrophobic, leaderless or denatured proteins, or hydrophobic signaling molecules (for recent reviews, see Jurkoshek *et al.*^2016^; Penfornis *et al.*^2016^; Tkach and Théry 2016; Domingues and Nielsen 2017; Kouwaki *et al.*^2017^; Takahashi *et al.*^2017^; Toyofuku *et al*. 2017a). Additionally, the transfer of nucleic acids between cells via EVs is being increasingly investigated. In Archaea and Bacteria, fusion of EVs carrying DNA between cells has been proposed as a novel mechanism for horizontal gene transfer (HGT), in addition to the well-known mechanisms of transformation, transduction and conjugation (Domingues and Nielsen 2017; Erdmann *et al.*^2017^). In Eukaryotes and Bacteria, EVs seem to be involved in the long-distance transfer of genetic information via the shuttling of regulatory or mRNA between cells (Tsatsaronis *et al.*^2018^).

The impact of EV production on pathogenesis is becoming an increasingly active research field (El Andaloussi *et al.*^2013^). It is now well established that many pathogenic bacteria use their EVs to deliver toxic compounds to infected cells (Bitto *et al.*^2017^) whereas eukaryotic EVs are involved in many important human pathologies, from cancer to cardiovascular and neurodegenerative diseases with many laboratories exploring their potential to be used as biomarkers or delivery vehicles for therapeutic action (Yáñez-Mó *et al.*^2015^; Robbins, Dorronsoro and Booker 2016; Thompson *et al.*^2016^; Liu *et al.*^2017^; Mateescu *et al.*^2017^). EVs are also major players in aging (Takasugi 2018). Remarkably, the composition of EVs changes with age in humans, and a pioneering experiment demonstrated that administration of EVs isolated from young cells ameliorates age-related functional decline in older mice (Zhang *et al.*^2017^).

**Figure 3.**
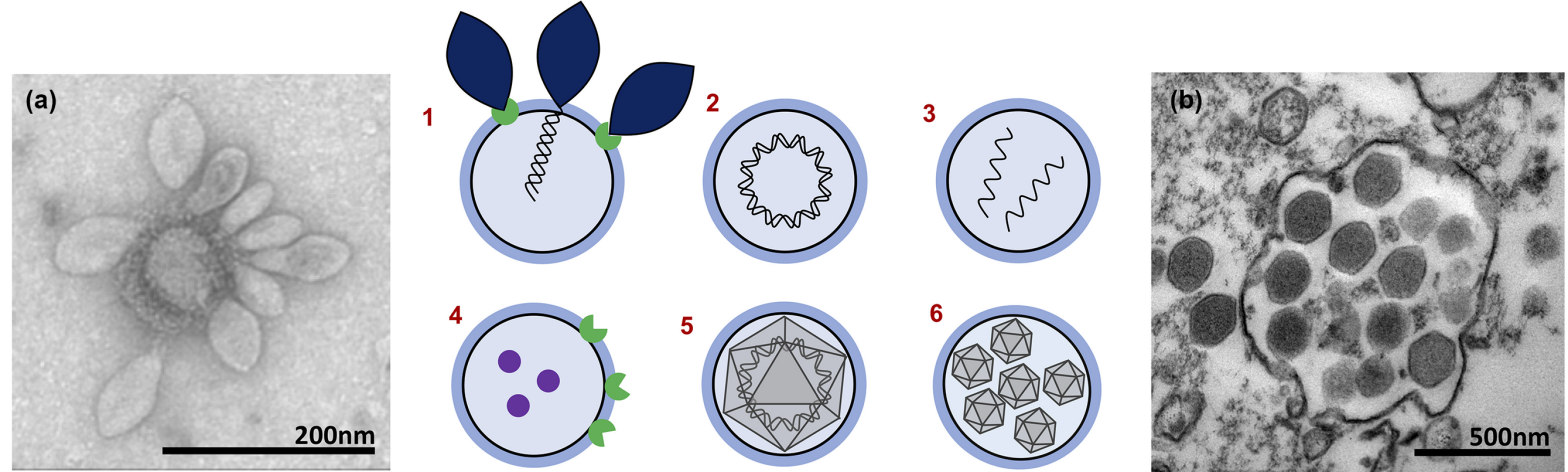
EVs and viruses interact in multiple ways. **1** and (**a**)**:** Virus receptors on vesicles could act as decoys protecting the host from infection. **(a)** TEM showing several *Sulfolobus* spindle-shaped virus 1 (SSV1), from the *Fuselloviridae* family, attached to a membrane vesicle. **2** and **3:** Encapsulated DNA/ RNA can be infectious as in pleolipoviruses or plasmidions. **4:** Virus receptors and effectors can transfer between cells, promoting infection of non-susceptible hosts. **5:** Membrane-bound viruses resist human attack. **6** and (**b**): VPVs allow for high MOI and 'Trojan horse’-style infection. Image (a) kindly provided by Virginija Krupovic, Institut Pasteur, France. Image (b) kindly provided by Jônatas Santos Abrahão, Institute of Biological Sciences, Universidade Federal de Minas Gerais, Brazil and obtained by the Center of Microscopy of UFMG, Brazil.

A research area which has become increasingly important in recent years is that of the interactions between EVs and viruses. Strikingly, EVs resemble the virions of enveloped viruses when observed by electron microscopy. Furthermore, EVs can attach to virions (if EVs harbor virus receptors at their surface), engulf viral particles or mimic viral particles by carrying viral proteins, RNA and DNA (Fig. 3). Some EVs containing viral genome or plasmids have been described as viral or plasmid vesicles (plasmidions) and could facilitate the propagation of these mobile elements (Forterre, Da Cunha and Catchpole 2017). Whereas EVs can sometimes act as decoys to limit viral infection, virus themselves can manipulate the production of EVs from infected cells (the virocell, *sensu* Forterre 2013) to their own benefit (Altan-Bonnet 2016). These observations have fueled speculation on the physiological and/or evolutionary relationships between EVs and viruses, suggesting that studying EVs could be helpful in understanding the origin of viruses themselves (Jalasvuori and Bamford 2008; Forterre and Krupovic 2012).

Finally, the ubiquity of EVs suggests that their production could have already existed at the time of the last universal common ancestor (LUCA) (Gill and Forterre 2016). However, it remains to be seen if any of the modern mechanisms of EV production are homologous in the three domains of life, testifying for their antiquity, or if different mechanisms of EV production have originated independently in different domains. Unfortunately, our knowledge concerning the mechanisms of EV biogenesis is still very limited, and as yet it has not been possible to draw clear-cut evolutionary connections between their modes of production in different domains. Genetic and biochemical analyses have only begun to elucidate mechanistic aspects of EV production in Bacteria (Wessel *et al.*^2013^; Devos *et al.*^2015^; Kulp *et al.*^2015^; Roier *et al.*^2015^, 2016a,b; Resch *et al.*^2016^; Turnbull *et al.*^2016^; Ojima *et al.*^2017^) and Eukaryotes (Muralidharan-Chari *et al.*^2009^; Ostrowski *et al.*^2010^; Oliveira *et al.*2010b; Gross *et al.*^2012^; Rilla *et al*. 2013, 2014; Tricarico, Clancy and D'Souza-Schorey 2016). These analyses suggest that membrane protein/lipid composition play a crucial role in EV production (Bonnington and Kuehn 2016; Elhenawy *et al.*^2016^; Skotland, Sandvig and Llorente 2017).

All these fascinating new discoveries and hypotheses explain why, in recent years, specialized journals focusing on EVs have been launched, such as *the Journal of Extracellular Vesicles*, and regular international meetings dedicated to EVs have been established as well as an academic society, *the International Society for Extracellular Vesicles* (ISEV). The data from various EV studies have been listed in three databases dedicated to EVs, namely Exocarta (lipids, RNA and proteins identified in exosomes), Vesiclepedia (data from different types of EVs) and EVpedia (high-throughput analyses and data on proteins, nucleic acids and lipids EVs) (Mathivanan and Simpson 2009; Kalra *et al.*^2012^; Kim *et al*. 2013, 2015).

**Figure 4.**
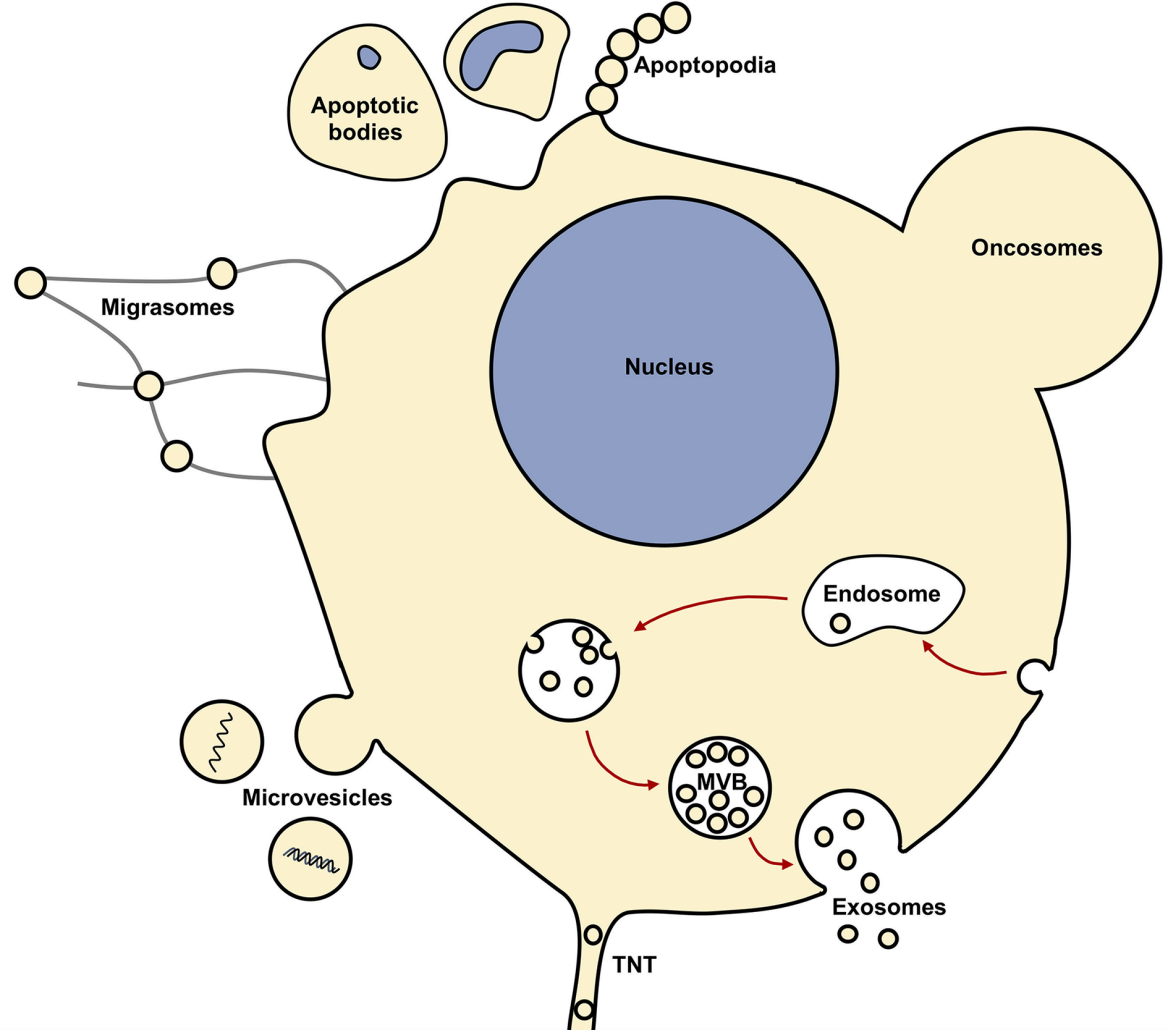
EV production in Eukaryotes. Multiple types of EVs originate through many complex and varied pathways. Eukaryotic EV functions include protein sorting/trafficking, intercellular communication, host adaptation during infection, metastatic niche adaptation, immune evasion and pathogenesis.

EVs are diverse in origin and nature, and there is little consensus on their classification (Gould and Raposo 2013). They are known under a variety of names such as membrane vesicles, extracellular membrane vesicles, microvesicles, microparticles, exosomes and ectosomes (in Eukaryotes), as well as more specialized names for particles arising from specific cell types e.g. oncosomes (produced by cancer cells), migrasomes (produced by amoeboid cells), apoptotic bodies (produced by cells during apoptosis), etc. (Colombo, Raposo and Théry 2014; Minciacchi *et al.*^2015^) (Fig. 4). In recent years, the term ‘extracellular vesicles’ (EV) has been regularly used in most reviews on this topic. This term has been adopted by ISEV and journals devoted to their studies (Mateescu *et al.*^2017^). Throughout this review, the term ‘extracellular vesicles’ (EVs) will be used *a priori* to refer to all types of membrane vesicles in the three domains of life, except when the identification of a specific subset of EVs is well documented, such as the well-known outer membrane vesicles (OMV) produced by Bacteria.

The number of excellent reviews discussing recent and past studies on EVs has exploded of late. Many of them have focused on particular area of EV studies such as HGT (Domingues and Nielsen 2017), immune response regulation (Kouwaki *et al.*^2017^), aging (Takasugi 2018), RNA content (Tsatsaronis *et al.*^2018^), EVs and cancer (Xu *et al.*^2018^) or interactions between EVs and viruses (Altan-Bonnet 2016; Nolte-‘t Hoen *et al.*^2016^). Some have covered EVs produced by Bacteria, especially dealing with OMVs (Schwechheimer and Kuehn 2015; O’Donoghue and Krachler 2016; Jan 2017; O’Donoghue *et al.*^2017^; Toyofuku *et al*. 2017a) but most of them are devoted to eukaryotic EVs, with an emphasis on exosomes (Yáñez-Mó *et al.*^2015^; Kalra, Drummen and Mathivanan 2016; Guo *et al.*^2017^; Rashed *et al.*^2017^; De la Torre Gomez *et al.*^2018^). In a review sponsored by the European COST action initiative ‘Microvesicles and Exosomes in Disease and Health’, the authors describe in great detail EVs in ‘higher’ and ‘lower’ organisms (Eukaryotes) and devote only a tenth of this review to EVs from pathogenic bacteria, without a single word on EVs produced by Archaea (Yáñez-Mó *et al.*^2015^).

The traditional divide between prokaryotes and eukaryotes has profoundly influenced biologists; bacterial EVs have been studied independently of eukaryotic ones, and archaeal EVs for a long time have been mostly ignored. Among eukaryotes, most studies on EVs have focused on human EVs, often without realizing that this was not a specific phenomenon restricted to multicellular ‘higher’ organisms, and that studying this process in other model organisms, including microorganisms, could provide new insights that could be useful to grasp its generality and specificity. Here, we review EV biology and discuss various roles EVs play in the three domains of life, with some emphasis on archaeal EVs (to compensate for their absence in many other reviews) and on the interactions between EVs and the viral world, a research area in which connections between discoveries made in different domains of life is especially striking. We also briefly discuss the possible role of EVs at different steps of cellular evolution, in particular regarding the role of EVs in recent hypotheses on the origin of Eukaryotes (Baum and Baum 2014; Gould, Garg and Martin 2016). We hope that the comparative approach used in this review will help to make the study of EVs a common goal shared by all biologists.

### EVs in Bacteria

The domain Bacteria contains very diverse prokaryotic microorganisms unified by common informational machineries for DNA replication, transcription and translation that are strikingly different from those of Eukaryotes and Archaea (Woese, Kandler and Wheelis 1990). Bacteria are also characterized by the presence of peptidoglycan in their cell wall, a rigid biopolymer that creates conditions for EV production quite distinct from those in the other two domains. Peptidoglycan was probably already present in the last bacterial common ancestor and is only lacking today in a few bacterial groups (Mycoplasmatales and some Planctomycetales). Bacteria exhibit various types of cell envelopes that will impact on the nature of their EVs. The vast majority of Bacteria have an outer lipopolysaccharide (LPS)-containing membrane and a rather thin layer of peptidoglycan located in the periplasmic space, i.e. between the outer and inner membrane. They are usually referred to as Gram-negative or diderm bacteria. In contrast, most bacteria of the phylum Firmicutes stain Gram positive and are sometimes referred as monoderm bacteria because they have a single membrane covered by a thick layer of peptidoglycan. Bacteria of the phylum Actinobacteria, including such important species as *Streptomyces* and Mycobacteria, are rather distinct from both the classical monoderms and diderms. They stain Gram positive because their thin peptidoglycan is directly covered by a thick polysaccharide layer. Many bacteria also contain a proteineous S-layer that plays an important role in the interaction between bacteria and their environment (Fagan and Fairweather 2014). However, this S-layer has been lost in many species that have been studied for a long time in the laboratory, especially those that have been studied for EV production.

**Figure 5.**
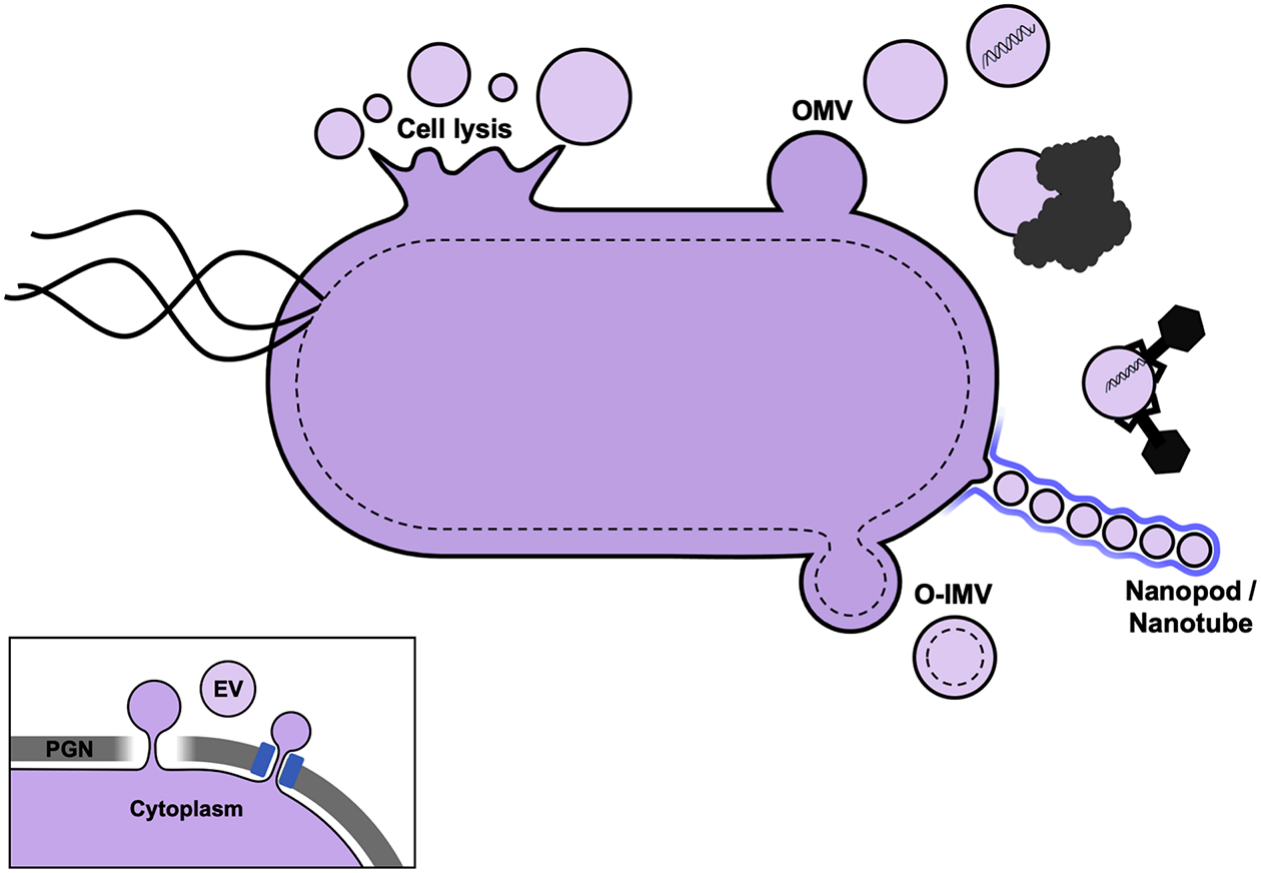
EV production in Bacteria. Two main types of EVs originate from diderm bacteria (OMVs and O-IMVs); however, cell lysis and nanotubes also produce EVs. Functions include intercellular communication, HGT, biofilm formation/maintenance, biomineralization, pathogenesis, viral defense, disposal/detoxification and relief of envelope stress. Inset: EVs in Firmicutes are produced from the single cytoplasmic membrane and must cross the thick PGN layer either by degradation of PGN or through pores.

**Figure 6.**
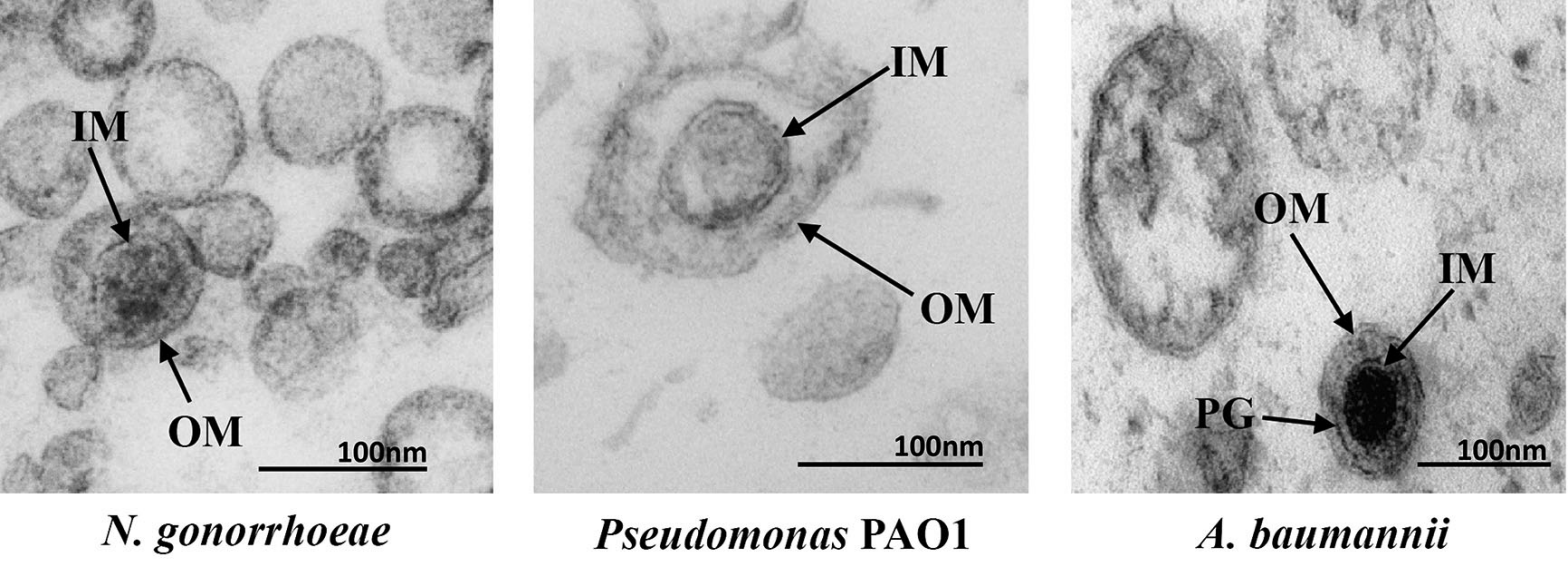
TEM of ultrathin sections of EVs from three bacterial species. Both OMVs and O-IMVs are observed in these EV preparations, with features of O-IMVs indicated. O-IMVs are surrounded by an external bilayer, probably corresponding to the outer membrane (OM) of the cell, and an inner membrane (IM), probably corresponding to the cytoplasmic membrane, which entraps electron dense material. In the image of O-IMVs from *A. baumannii*, the putative peptidoglycan layer (PG) can be seen. Images kindly provided by Elena Mercade and Carla Pérez-Cruz (Universitat de Barcelona, Spain).

The majority of EV studies in Bacteria have been carried out on Proteobacteria, the most abundant and well-studied phylum of diderm bacteria. Early studies focused on model organisms and/or pathogenic species of Proteobacteria such as *Escherichia coli*, *Neisseria meningitidis*, *Pseudomonas aeruginosa*, *Shigella flexneri*, *Helicobacter pylori, Legionella pheumophila and Shewanella livingstonensis* (for early publications, see reviews by Beveridge 1999; Mashburn-Warren and Whiteley 2006; Kulp and Kuehn 2010 and references therein). The study of bacterial EVs was mainly pioneered by studies in the laboratory of Terry Beveridge at a time when this phenomenon was still largely underestimated by microbiologists (Kadurugamuwa and Beveridge 1995; Beveridge 1999). Several types of EVs have been described in Bacteria, with the most studied being the so-called OMVs produced by diderm bacteria. These OMVs are formed by budding of the LPS-containing outer membrane (OM) and mainly contain periplasmic components (Figs 5 and 6). However, EVs containing both the outer and inner membranes of diderm bacteria have been recently identified in several species, and termed outer-inner membrane vesicles (O-IMV) (Pérez-Cruz *et al.*^2013^, ^2015^) (Fig. 6). These EVs contain both periplasmic and cytoplasmic components and originate from the cytoplasmic membrane. EVs have also been observed from monoderm bacteria with thick cell walls, such as Firmicutes and Actinobacteria (Prados-Rosales *et al.*^2014^). This was surprising, since it was expected that EVs could not escape such large barriers. How these EVs traverse the wall is, as yet, unknown (Brown *et al.*^2015^). Finally, it is increasingly appreciated that bacterial EVs are heterogeneous populations of EVs with various size, density and cargo content, whose production and relative distribution change with the physiological state (Dauros Singorenko *et al.*^2017^). The current challenge is to develop methods allowing to reproducibly analyze specific types of EVs.

The production of EVs by Bacteria is not an artifact of laboratory culture conditions. Indeed, Bacteria have been shown to produce EVs in biofilms and during infections (Schooling and Beveridge 2006; Marsollier *et al.*^2007^; Deatherage and Cookson 2012; Schwechheimer and Kuehn 2015). EVs in biofilms interact with extracellular DNA to reinforce structural integrity and to also serve as decoys against antibiotics (Schooling, Hubley and Beveridge 2009). For a long time, the presence of EVs in natural environments was largely ignored. A seminal study by Biller and co-workers highlighted the abundance of bacterial vesicles, from the marine phototrophic bacteria *Prochlorococcus*, in marine ecosystems as well as in the laboratory (Biller *et al.*^2014^). Importantly, they showed that *Prochlorococcus* vesicles can be used as food, supporting the growth of heterotrophic bacterial cultures, suggesting that EVs also impact the marine carbon flux. The authors succeeded in isolating EVs from two very different ocean samples, with concentrations ranging from 10^5^ to 10^6^ vesicles per ml of sea water.

### Outer membrane vesicles produced by diderm bacteria

Most EVs produced by diderm bacteria derive from the OM and are referred to as OMVs (for recent reviews, see Schwechheimer and Kuehn 2015, Orench-Rivera and Kuehn 2016; Jan 2017; Toyofuku *et al*. 2017b). During growth, the OM ‘blebs’ outwards and pinches off, forming spherical vesicles (20–250 nm) derived from the OM and trapped periplasmic components (Fig. 5). OMVs thus contain components of the OM and periplasm, such as bacterial lipids, OM proteins, soluble proteins, integral membrane proteins, lipoproteins and glycolipids. In fact, the identification of LPS and OM proteins was used to confirm their OM origin. Mass spectrometry-based proteomic studies were used to characterize the protein components of OMVs (Lee *et al.*^2008^; Choi *et al.*^2011^; Altindis, Fu and Mekalanos 2014; Jang *et al.*^2014^; Kieselbach *et al.*^2015^; Lee, Kim and Gho 2016; Yun *et al.*^2017^). Although cytoplasmic proteins were believed to be depleted in OMVs (Kulp and Kuehn 2010), some proteomic studies demonstrated their presence, despite following stringent purification protocols (Pérez-Cruz *et al.*^2013^; Berleman *et al.*^2014^). Further studies are necessary to understand why these cytoplasmic proteins would be associated with OMVs and what role they play (Schwechheimer and Kuehn 2015).

Increased vesiculation may be a response to stress and aid the removal of toxic by-products after exposure to stressful conditions (McBroom and Kuehn 2007; Maredia *et al.*^2012^; Macdonald and Kuehn 2013). For example, exposure of *Stenotrophomonas maltophilia* to the antibiotic ciprofloxacin resulted in an increased release of heterogeneous OMVs (Devos *et al.*^2017^).

OMVs can deliver their cargoes to recipient bacteria and have been implicated in inter- and intracellular communication, biofilm formation (Liao *et al.*^2014^; Turnbull *et al.*^2016^), antibiotic resistance (Rumbo *et al.*^2011^), stress response (Maredia *et al.*^2012^), toxin delivery (Rompikuntal *et al.*^2012^) and the transfer of nucleic acids (Biller *et al.*^2014^; Ghosal *et al.*^2015^; Sjöström *et al.*^2015^; Blenkiron *et al.*^2016^; Koeppen *et al.*^2016^; Resch *et al.*^2016^; Bitto *et al.*^2017^; Domingues and Nielsen 2017; Tsatsaronis *et al.*^2018^; Dauros-Singorenko *et al.*^2018^) (Fig. 5). In addition, OMVs can also deliver their cargoes to eukaryotic cells and have been implicated in pathogenesis (delivery of toxins and virulence factors) and homeostasis of the immune system (Nakao *et al.*^2011^; Lee *et al.*^2012^; Elhenawy, Debelyy and Feldman 2014; Rakoff-Nahoum, Coyne and Comstock 2014; Hickey *et al.*^2015^; Muraca *et al.*^2015^; Celluzzi and Masotti 2016; Jan 2017). For instance, *Bacteroides thetaiotaomicron* OMVs cross can be detected within gut mucosal macrophages suggesting that they can cross the mucosal barrier and initiate intestinal inflammation (Hickey *et al.*^2015^; Pathirana and Kaparakis‐Liaskos 2016).

Besides phagocytosis, four routes of OMV uptake have been implicated in host cells. These include macropinocytosis; clathrin-mediated endocytosis; caveolin-mediated endocytosis or non-caveolin, non-clathrin mediated endocytosis (Rewatkar *et al.*^2015^; O’Donoghue and Krachler 2016). For more details, refer to the reviews by Villarroya-Beltri *et al.*^2014^; Kaparakis-Liaskos and Ferrero 2015; O’Donoghue and Krachler 2016; Bitto *et al.*^2017^). A recent study using a genetically encoded OMV probe and cell permeable dye showed that entry and efficiency of uptake are influenced by the bacterial cell wall composition (O’Donoghue *et al.*^2017^).

OMVs are resistant to attack by degradative enzymes thus enabling long distance delivery, specificity in host-cell targeting and evasion of the host immune system (Bonnington and Kuehn 2014). As an example, OMVs of *Moraxella catarrhalis* help cells evade the immune system by bearing antigens which serve as decoys (Tan *et al.*^2007^*;* Perez Vidakovics *et al.*^2010^). OMVs can also help in bacterial colonization by selectively killing or promoting the growth of other bacteria species (Kadurugamuwa and Beveridge 1996; Ellis and Kuehn 2010; Hickey *et al.*^2015^).

OMVs are a powerful and versatile tool for alternative vaccination strategies due to their immunogenic properties, natural adjuvanticity, uptake by mammalian cells and potential for genetic engineering (van der Pol, Stork and van der Ley 2015). These properties have already been exploited to develop two meningitis vaccines with components from the OM and periplasm of *N. meningitidis* (Holst *et al.*^2009^; Granoff 2010). OMVs can be glycoengineered (geOMVs) to display surface glycans of the pathogen of interest. These geOMVs have been shown to be efficient against *Streptococcus pneumoniae* in mice and against *Campylobacter jejuni* in chickens (Price *et al.*^2016^). One of the main drawbacks of vesicle-based vaccines is the presence of LPS lipid A, which elicits a strong inflammatory response in the host. Using the glycoengineering approach it may be possible to modify LPS lipid A, thereby reducing its toxicity. To fully exploit OMV-based vaccines, novel genetic tools are needed to load the desired recombinant antigens onto the OMVs. For a recent review on the therapeutic potential of bacterial vesicles, refer to Bitto and Kaparakis-Liaskos (2017).

### Outer-inner bacterial EVs

Bacterial EVs containing both the outer and cytoplasmic membrane were recently described for the Antarctic bacterium *S*. *vesiculosa* M7T. These double-membrane vesicles are known as ‘outer-inner membrane vesicles’ (O-IMVs) (Pérez-Cruz *et al.*^2013^). Similar EVs were later observed in cultures of diderm pathogenic bacteria such as *N. gonorrhoeae*, *P. aeruginosa* PAO1 and *Acinetobacter baumannii* AB41 (Fig. 6) (Pérez-Cruz *et al.*^2015^). As in the case of OMVs, it was suggested that these O-IMVs may be involved in lateral gene transfer and the transfer of cytoplasmic proteins. As O-IMVs are formed by the protrusion of both the outer and cytoplasmic membranes (Fig. 5), they should be enriched in cytoplasmic components, such as DNA and ATP, relative to OMVs. Very little is known about the production mechanism of these O-IMVs, especially with regard to differences between these and classical OMVs. Recently, it has been observed that the diderm bacterium *S. maltophilia* produced both OMV and O-IMVs following ciprofloxacin induction (Devos *et al.*^2017^). In fact, it is possible that most bacteria-producing OMVs also produce O-IMVs to some extent and that O-IMVs have been understudied for methodological reasons and also due to the fact that attention was focused on OMVs.

### EVs in Firmicutes

Despite their thick peptidoglycan layer, monoderm Firmicutes also produce EVs. The first hint of the presence of EVs in Firmicutes came from studies with *Bacillus subtilis* and *B. cereus* (Dorward and Garon 1990). EVs from *B. subtili*s are heterogeneous and their diameter correlates with electron density, suggesting that cargo selection and vesicle size may be linked (Brown *et al.*^2015^). Following their first observation, several studies have purified EVs from the supernatants of cultured Firmicutes (Klieve *et al.*^2005^; Lee *et al.*^2009^; Rivera *et al.*^2010^; Jiang *et al.*^2014^; Olaya-Abril *et al.*^2014^; Prados-Rosales *et al.*^2014^; Brown *et al.*^2015^; Resch *et al.*^2016^). In most Firmicutes, the size range of EVs falls within 20 nm–150 nm in diameter. However, some species such as *Bacillus* spp. and *Clostridium perfringens* produce larger EVs (up to 400 nm) (Brown *et al.*^2015^).

Two non-exclusive hypotheses have been proposed to explain how EVs can cross the large barrier of the peptidoglycan. It was suggested that EVs may be forced through pores in the cell wall by turgor pressure after budding from the cell membrane and/or that the peptidoglycan was locally destroyed by enzymes associated with EVs and/or released with EVs (Brown *et al.*^2015^). This second hypothesis was supported by the presence of peptidoglycan-degrading enzymes in EVs isolated from *Staphylococcus aureus* (Lee *et al.*^2009^). It has also been reported that a subpopulation of *B. subtilis* expresses a prophage-encoded endolysin that degrades peptidoglycan allowing EV release (Toyofuku *et al.*2017b). More recently, it has been demonstrated that *S. aureus* produce enzymes, such as phenol-soluble modulins and autolysins, that are implicated in facilitating EV formation (Wang *et al.*^2018^).

The membrane and the lumen of EVs from Firmicutes are thought to be derived from the cytoplasmic membrane and the cytoplasm, respectively (MacDonald and Kuehn 2012). These EVs can thus transport a variety of cargoes, including cytoplasmic material, such as RNA and DNA (see below). The protein cargo identified in EVs from Firmicutes includes enzymes involved in peptidoglycan degradation, antibiotic degradation, virulence factors (e.g. anthrolysin, anthrax toxin components, coagulases, hemolysins and lipases) and immunologically active compounds (Marsollier *et al.*^2007^; Lee *et al.*^2009^; Rivera *et al.*^2010^; Gurung *et al.*^2011^; Prados-Rosales *et al.*^2011^; Thay, Wai and Oscarsson 2013; Brown *et al.*^2014^; Resch *et al.*^2016^; Vdovikova *et al.*^2017^). Proteomic approaches have been used to characterize EVs produced by Firmicutes (Lee *et al.*^2009^, ^2013^; Resch *et al.*^2016^). For example, proteomic analysis of *Listeria monocytogenes* EVs revealed that they were enriched in proteins important for survival and virulence, including the hemolysin listeriolysin O (LLO) (Lee *et al.*^2013^), whereas protein analysis of EVs produced by Group A *Streptococcus* revealed both unique proteins and proteins specifically enriched in EVs among the 195 proteins identified in the EV proteome (Resch *et al.*^2016^).

A recent study demonstrated that EVs carrying LLO offered a protective effect against autophagy and cell death (Vdovikova *et al.*^2017^). Many Firmicutes are important and well-studied pathogenic species, and many lines of evidence suggest that the EVs produced by Firmicutes are also involved in pathogenesis (Lee *et al.*^2009^; Rivera *et al.*^2010^; Gurung *et al.*^2011^; Prados-Rosales *et al.*^2011^; Thay, Wai and Oscarsson 2013; Pathirana and Kaparakis‐Liaskos 2016; Resch *et al.*^2016^; Vdovikova *et al.*^2017^).

### Bacterial EVs as DNA transfer agents

Many studies have now described the association of DNA with bacterial EVs, including OMV, O-IMVs and EVs produced by Firmicutes (Domingues and Nielsen 2017). The possibility that these EVs could be involved in lateral gene transfer has important implications for the transfer of antibiotic resistance and virulence genes but also more generally for bacterial evolution.

The first description of DNA associated with bacterial EVs was published in 1983 by Nobel laureate Hamilton Smith and colleagues, who described EVs produced by *Haemophilus influenzae* as ‘specialized membranous structures that protect DNA during *Haemophilus* transformation’ (Kahn, Barany and Smith 1983). The authors noticed that these so-called transformasomes protected DNA from DNase or restriction enzymes and can therefore constitute a new tool for HGT. Microbiologists remained rather skeptical towards this possibility for a long time. Later, Yaron *et al.* (2000) reported that EVs produced by the pathogenic strain *E. coli* O157:H7 can indeed mediate the transfer of virulence genes to other Enterobacteria. It is still unclear how DNA can be packaged into OMVs, which *a priori* only contain periplasmic components. It is often assumed that DNA is packaged in a subpopulation of ‘secondary’ OMVs formed by aggregation of cell wall fragments after cell lysis. In fact, the packaging of DNA within OMVs has not always been conclusively demonstrated (Renelli *et al.*^2004^; Mashburn-Warren and Whiteley 2006; Kulp and Kuehn 2010; Pérez-Cruz *et al.*^2013^; Liao *et al.*^2014^) and it is often not clear whether the nucleic acids are inside the vesicles or simply associated with the vesicles in such a manner where they resist enzymatic degradation (Domingues and Nielsen 2017). For instance, Pérez-Cruz *et al.* (2015) did not detect DNA in OMVs from *N. gonorrhoeae*, whereas they found DNA in O-IMVs produced by this bacterium (see below).

Biller and co-workers recently examined the quantity and distribution of DNA associated with OMVs from different bacteria. Their results demonstrated that the size and quantity of DNA varied amongst the different bacteria and that only a small proportion of EVs contain DNA (Biller *et al.*^2017^). Ferrero and colleagues show that most of the DNA associated with OMVs produced by *P. aeruginosa* is present on the external surface, with a smaller amount located inside OMVs (Bitto *et al.*^2017^). This DNA is mostly present in small OMVs of around 20 nm. The authors found that the external DNA includes fragments from the bacterial chromosome, whereas internal DNA was mainly composed of short fragments (1–4 kb) enriched in specific regions encoding proteins involved in virulence, stress response and antibiotic resistance.

Interestingly, studying OMVs from *Thermus* species, Blesa and Berenguer (2015) have suggested that EVs could function as reservoir of genetic material resistant to heat denaturation for transformation in high temperature environments, as previously proposed for hyperthermophilic archaea (Soler *et al.*^2008^). Transfer of DNA mediated by OMVs produced by *Thermus* species is dependent on the competency of the recipient cells, suggesting that DNA is not delivered by fusion of OMV with host cells (Blesa and Berenguer 2015).

Remarkably, Ferrero and colleagues could demonstrate that *P. aeruginosa* OMVs can transfer their DNA into the nucleus of eukaryotic cells (Bitto *et al.*^2017^). They suggest that the internal DNA plays a role in cell-to-cell communication whereas DNA present at the surface of OMVs performs a different role, being important for biofilm formation and protection. The latter observation is supported by the upregulation of DNA associated with OMVs during biofilm formation in *S. mutans* (Liao *et al.*^2014^).

The presence of DNA in EVs from monoderm organisms was anticipated, considering that their EVs were expected to contain cytoplasmic components. The presence of DNA associated with EVs from Firmicutes and the capacity of these EVs to transfer genetic markers to recipient cells was first observed in *Ruminococcus* species (Klieve *et al.*^2005^). This DNA was described as short fragments of chromosomal DNA ranging from 23 to 90 kb. Interestingly, the authors reported that, unlike chromosomal DNA, EV-associated DNA was resistant to digestion with EcoRI, suggesting differences in the restriction/modification pattern of these DNAs.

### Bacterial EVs as RNA transfer agents

The discovery in the first decade of this century that eukaryotic EVs can deliver RNA cargoes to recipient cells, promoting phenotypic changes (see below), prompted several authors to search more recently for RNA associated with bacterial EVs (for review, see Dauros-Singorenko *et al.*^2018^; Tsatsaronis *et al.*^2018^). RNA was first detected in preparations of EVs produced by *Prochlorococcus*, and covered a remarkable 95% of the genome (Biller *et al.*^2014^). RNA was also detected associated with OMVs of *Vibrio cholerae* (Sjöström *et al.*^2015^), *E. coli* (Ghosal *et al.*^2015^; Blenkiron *et al.*^2016^), *P. aeruginosa* (Koeppen *et al.*^2016^) and in EVs of Group A *Streptococcus* (Resch *et al.*^2016^). The majority of RNA in bacterial EVs are rather short (less than 250 nucleotides) and resistant to RNase treatment. However, Sjöström and colleagues reported that RNA associated with *V. cholera* OMVs was sensitive to RNase, suggesting that it could be located at the surface of the OMVs. Alternatively, the authors suggested that RNAse could have entered these OMVs, impairing their integrity (Sjöström *et al.*^2015^). It is likely that, similarly to DNA, RNA could be located both inside and outside of the EVs.

In several studies, RNAs associated with EVs were analyzed using deep RNA-sequencing, revealing a large diversity of RNA molecules (Ghosal *et al.*^2015^; Resch *et al.*^2016^), including rRNA, tRNA, mRNA and a variety of small RNA species, including CRISPR RNAs guides (Resch *et al.*^2016^). Importantly, RNA associated with bacterial EVs can be delivered into the cytoplasm and nuclei of the host cell (Blenkiron *et al.*^2016^). Notably, RNA associated with *P. aeruginosa* OMVs can be transferred to infected human and mouse cells, decreasing their innate immune response (Koeppen *et al.*^2016^). Charpentier and colleagues thus propose that EVs are an important source of microbial RNAs that modulate the immune response during infection (Tsatsaronis *et al.*^2018^).

In general, it seems that mRNA is under-represented in EVs relative to the cellular RNA pool whereas RNAs originating from intergenic region are over-represented. However, Resch and colleagues reported that many mRNA species were present in Group A *Streptococcus* EVs and that some of them were specifically enriched in EVs. This suggests that EVs could trigger the production of new proteins in recipient cells.

### Biogenesis of bacterial EVs

Although OMVs have been observed for decades (Bishop and Work 1965; Work, Knox and Vesk 1966; Chatterjee and Das 1967), the process by which diderm organisms produce them is not fully understood. The enrichment or depletion of OMV content compared to the cell suggests that it is a deliberate and regulated process (Deatherage and Cookson 2012; Schertzer and Whiteley 2012; Schwechheimer and Kuehn 2015). To date, several models for OMV biogenesis have been proposed (Mashburn-Warren and Whiteley 2006; Kulp and Kuehn 2010; Haurat, Elhenawy and Feldman 2015; Roier *et al.*2016a,b). However, a conserved general mechanism for biogenesis has remained elusive for a long time (Roier *et al.*2016a,b). This is changing as genetic and biochemical analyses over the years have begun to shed light on aspects of OMV biogenesis in diderm bacteria. Several mutants have been isolated in different species with either hypo- or hypervesiculation phenotypes. For instance, transposon mutagenesis in *H. influenzae* and a whole-genome knockout library of *E. coli* implicated 20 and 150 new genes in the process of vesiculation, respectively (Kulp *et al.*^2015^; Roier *et al.*^2015^). Most of these mutations trigger an increase in OMV formation (hypervesiculation), whereas a few others trigger a decrease (hypovesiculation). Analyses suggest that mutations in OM structures result in hypervesiculation in agreement with several proposed models for OMV production, whereas mutations in oxidative stress response pathways showed a decrease in vesiculation, in agreement with the implication of OMV production in stress response.

Earlier models suggested that OMV formation occurs due to the presence of fewer covalent linkages between the OM and the underlying peptidoglycan layer (Hoekstra *et al.*^1976^), due to the OM growing faster in certain regions (Chatterjee and Das 1967; Wensink and Witholt 1981). As a result, the OM bulges out and vesicles are formed (McBroom, Johnson and Vemulapalli 2006; Kulp and Kuehn 2010; Roier *et al.*2016a,b). This model is supported by the fact that deletion or truncation of genes encoding OmpA (OMP5), an abundant protein linking the OM to the peptidoglycan, triggered hypervesiculation in *E. coli*, *V. cholera* and *Salmonella* (Sonntag *et al.*^1978^; Song *et al.*^2008^; Deatherage *et al.*^2009^).

A subsequent model proposed that accumulation of peptidoglycan fragments (Hayashi, Hamada and Kuramitsu 2002) and misfolded proteins (McBroom and Kuehn 2007) due to stress increases turgor pressure in the periplasm and leads to the OM bulging out (Zhou *et al.*^1998^; Roier *et al.*2016a,b). An increase in OMV production was indeed observed when peptidoglycan fragments accumulated because of the incomplete degradation of peptidoglycan in a *Porphyromonas gingivalis* autolysin mutant strain (Hayashi, Hamada and Kuramitsu 2002). Additionally, deletion of the *degQ* gene, which encodes a periplasmic serine protease in *S. oneidensis*, resulted in an increased level of protein accumulation within the periplasm and subsequently a hypervesiculation phenotype (Ojima *et al.*^2017^).

A third model has been proposed based on the importance of charge–charge interactions. The LPS composition in the OM can vary in response to various environmental factors. For example, *P. aeruginosa* possesses two distinct LPS O-polysaccharide species, A (neutral)- and B-band (charged) LPS. In wild-type cells, the OMVs are enriched in the B-band LPS, which creates repulsion by accumulation of negative charges and therefore leads to membrane protrusion (Kadurugamuwa and Beveridge 1995). Indeed, cells expressing the neutral A band LPS produce smaller OMVs (Murphy *et al.*^2014^).

Additionally, a bilayer-couple model for OMV biogenesis was proposed in *P. aeruginosa.* Insertion of Pseudomonas quinolone signal (PQS) into the outer leaflet of the OM can also increase membrane curvature and lead to the formation of OMVs (Mashburn-Warren and Whiteley 2006; Schertzer and Whiteley 2012; Florez *et al.*^2017^). Mutants lacking OprF (an OmpA homolog) in *P. aeruginosa* have increased levels of PQS and thus increased OMV production (Schertzer and Whiteley 2012; Wessel *et al.*^2013^). However, PQS is only produced by *P. aeruginosa*, and therefore this model is limited to this species.

A more general model was proposed based on studies performed on two distantly related Proteobacteria, *H. influenzae* and *V. cholerae* (Roier *et al.*2016a,b). Among hypervesiculation mutants obtained by transposon mutagenesis in *H. influenzae*, Roier and colleagues focused on mutants of the VacJ/Yrb ABC transporter system, which is widely conserved in Proteobacteria and known to prevent phospholipid accumulation in the outer leaflet of the OM. A similar hypervesiculation phenotype was found in *VacJ* and *Yrb* mutants of *V. cholerae*. The hypervesiculation phenotype of these mutants and biochemical analysis of the OMVs they produce suggest a model in which OMV formation is induced by the accumulation of phospholipids in the outer leaflet of the OM, thereby producing an asymmetric expansion and outward bulging of this membrane. Interestingly, Roier and co-workers observed that iron limitation induced by deletion of the *fur* gene (an activator of iron-regulated genes), a condition frequently observed for bacterial pathogens in their host, correlates with a downregulation of *vacJ/yrbB* genes and an increase in OMV production. They suggest that iron-limiting conditions in hosts result in increased OMV production by pathogenic bacteria, which bind antibodies and complement attacks, consistent with previously observed immune responses (Tan *et al.*^2007^*;.*Perez Vidakovics *et al.*^2010^). More generally, they propose that the asymmetric distribution of phospholipids between the inner and outer leaflets of the OM represents a general mechanism that can account for OMV formation under all growth conditions.

Homologs of proteobacterial VacJ and YrbB proteins are only present in a few bacterial phyla. It is thus likely that different mechanisms controlling phospholipid asymmetry between the inner and outer leaflets of outer and inner membrane phospholipid bilayers exist in different bacterial phyla. This suggests screening for such mechanisms and their potential role in OMV or OMV-IMV formation in Bacteria may enhance our understanding of EV production. Consistent with this hypothesis, a recent study proposed that LPS remodeling exerts modifications in the curvature of the OM leading to OMV formation (Elhenawy *et al.*^2016^). Deacylation of lipid A, the hydrophobic anchor of LPS, was examined in *Salmonella typhimurium*. Expression of the lipid A deacylase, PagL, resulted in hypervesiculation with the deacylated lipid accumulating exclusively in OMVs (Elhenawy *et al.*^2016^) (Fig. 1a). Additionally, a *ΔpagL* strain showed a significant reduction in OMV secretion relative to the wild-type strain. In another study, Bonnington and Kuehn suggested that OMVs are used by the cell to remove unfavorable LPS glycoforms. Thus, OMV production may aid in remodeling of the OM—a process essential to bacterial adaptation and survival in different niches (Bonnington and Kuehn 2016).

Finally, a recent study using super-resolution microscopy revealed that explosive cell lysis in *P. aeruginosa* can generate membrane fragments that rapidly form EVs (Turnbull *et al.*^2016^). This phenomenon is triggered by an endolysin encoded by a prophage integrated in the genome of *P. aeruginosa*. Notably endolysin-deficient mutants are defective in EV production and biofilm development. Thus, cell lysis could also act as a bona fide mechanism for the production of bacterial EVs. However, the extent to which this occurs has not been established. It is not clear whether it is possible to distinguish between ‘genuine’ and reconstituted EVs produced after cell lysis, even if the latter might have an extended subset of proteins, compared to EVs produced by other mechanisms.

### Cargo selection in OMVs

Selection of cargo is an important aspect of OMV biogenesis (Haurat *et al.*^2011^; Bonnington and Kuehn 2014; Tsatsaronis *et al.*^2018^). Evidence suggests that the cellular localization of a protein greatly affects its potential for inclusion into OMVs. Virulence factors such as alkaline phosphatase, phospholipase Cs, β-lactamase and Cif (CFTR inhibitory factor) are enriched in *P. aeruginosa* OMVs (Kadurugamuwa and Beveridge 1995; Bomberger *et al.*^2009^; Koeppen *et al.*^2016^). The loading of such virulence factors into vesicles is thought to rely on LPS subtypes. Proteins associated with charged LPS are enriched in OMVs, whereas those that co-localize with neutral LPS are retained in the OM (Haurat *et al.*^2011^; Veith *et al.*^2014^; Schwechheimer and Kuehn 2015).

Compared to OMVs, our knowledge about EV biogenesis in the Firmicutes is still in its infancy. Interestingly, Resch and colleagues discovered that production of EVs was negatively regulated by the two-component CovRS regulatory system, suggesting that CovRS could decrease EV production by triggering the expression of factors disrupting EV or preventing their release (Resch *et al.*^2016^). Resch and colleagues further observed that the phospholipid composition differs between EVs and the cytoplasmic membrane, with enrichment of phosphatidyl glycerol relative to cardiolipin, which is known to induce membrane curvature (Barák and Muchová 2013). They also reported an increase in monounsaturated fatty acid content. This indicates that modification of membrane lipid composition could play a critical role in EV production by Firmicutes, as in the case of OMVs by diderm bacteria.

### Nanotubes in Bacteria

In the last six years, several laboratories have found that diverse types of bacteria produce filamentous structures called nanopods or nanotubes that are involved in cell-to-cell transfer and seem intimately connected to EVs (for a recent review, see Baidya *et al.*^2018^). Similar filamentous structures containing EVs were previously observed in the culture medium of some hyperthermophilic archaea (Soler *et al.*^2008^) (see below) and resemble eukaryotic ‘tunneling nanotubes’ (Fig. 2) (Rustom *et al.*^2004^; Lou *et al.*^2012^). The first observation of a direct connection between EVs and nanotubes in Bacteria was reported for *Delftia acidovorans* that produces chains of EVs that are enclosed by the S-layer forming the typical structure referred to as nanopods (Shetty *et al.*^2011^). Similar structures, usually called Nanotubes, were then reported in Firmicutes (Dubey and Ben-Yehuda 2011, Dubey *et al.*^2016^), Myxobacteria (Ducret *et al.*^2013^; Remis *et al.*^2014^; Wei *et al.*^2014^) and Proteobacteria (*Francisella novicida*, *A. bayeli*, *E. coli*, *S. oneidensis*) (McCaig, Koller and Thanassi 2013; Pirbadian *et al.*^2014^; Pande *et al.*^2015^). In Firmicutes, the membranes of nanopods correspond to an extrusion of the cytoplasmic membrane that crosses the thick peptidoglycan layer, whereas they seem to be formed by extrusion of the OM in Proteobacteria and Myxobacteria.

These similar structures, thereafter called nanotubes, can bridge neighboring cells together either between the same or different species facilitating cell-to-cell communication (see below). Remarkably, it has been shown that the so-called nanowire filaments involved in the extracellular transport of electrons produced by *S. oneidensis* were in fact nanotubes associated with OMVs (Pirbadian *et al.*^2014^). In Myxobacteria, it has been shown that nanotube formation increases when cultures are grown without agitation (Wei *et al.*^2014^) and is upregulated in biofilms (Remis *et al.*^2014^). It is most likely that nanotubes are not laboratory curiosities but a fundamental mechanism for bacterial communication in nature. ‘Nanotubes’ appear either as purely tubular structures or as chains of consecutive constricted segments resembling EVs but having a continuous lumen. In contrast, ‘nanopods’ contain chains of discrete EVs. The two types of structures are sometimes produced by the same species (Dubey *et al.*^2016^) and studies with *Myxococcus xanthus* suggest that they could be in fact different states of the same kind of structure (Wei *et al.*^2014^). These differences could be also due to the method used for nanotube preparation and visualization. Hence, Jensen and co-workers only observed nanotubes formed by chains of OMVs when they analyzed the ultrastructure of nanotubes from *S. oneidensis* by electron cryotomography, whereas they appeared smooth in fluorescence light microscopy (Subramanian *et al.*^2018^). In this work, the authors observed electron dense region at the junction connecting neighboring OMVs, suggesting the existence of some unknown material that facilitates nanotube formation.

Nanotubes from Firmicutes have been studied quite extensively by the group of Ben-Yehuda. These authors reported that nanotubes can bridge neighboring *B. subtilis* cells as well as *B. subtilis* and *S. aureus* (Dubey and Ben-Yehuda 2011). They visualized a transfer of cytoplasmic fluorescent molecules between adjacent cells and reported that plasmids can be transferred from cell to cell via these nanotubes. Additionally, their work suggests these nanotubes can deliver toxins from *B. subtilis* to other bacilli, and following toxin delivery, the nanotubes can even facilitate ‘looting’, importing nutrients from the competitor cell (Stempler *et al.*^2017^). These nanotubes are formed even when the cells were grown to a low density and this production of extensive elongated nanotubes greatly increases the cell surface area (Dubey *et al.*^2016^). Utilizing a combination of super-resolution, light and electron microscopy, they described nanotubes as chains of membranous segments with a continuous lumen. Importantly, Ben-Yehuda and colleagues could detect in nanotubes of *B. subtilis* a calcineurin-like protein, YmdB, which is required for both nanotube formation and intercellular molecular exchange (Dubey *et al.*^2016^; Stempler *et al.*^2017^). The protein YmdB, a putative sensor phosphodiesterase involved in AMPc regulation, could be involved in transmitting messages for nanotube production by an unknown mechanism (Dubey *et al.*^2016^). The YmdB protein is only present in Bacteria but highly conserved among bacteria and present in several phyla, implying that it plays a fundamental role in bacterial physiology. It will be interesting to test if this protein is also involved in nanotube formation in different species.

Observations in real time showed that nanotubes produced by *B. subtilis* are formed very rapidly (in the course of minutes) and display rapid movements (Dubey *et al.*^2016^). Similar observations were made with nanotubes from *S. oneidensis* that were described as dynamic structures capable of growth, shrinking and reversible transition between OMV chain and individual vesicle morphology (Subramanian *et al.*^2018^).

### Intracellular vesicles in Bacteria

In Eukaryotes, some EV production is tightly linked to the network of intracellular vesicles that is typical of eukaryotic cells (see below). Although such intracellular vesicles are not as well known, nor as ubiquitous in Bacteria, they have been sometimes observed either within the cytoplasm or accumulating in the periplasm, see for example spectacular pictures in Dobro *et al.* (2017). Their function within the cell and their relationship with EVs remain unclear. Some intracellular vesicles seem to be involved in sequestration and detoxification of otherwise harmful compounds. For instance, it was recently shown that some sponge-associated bacteria mineralize both arsenic and barium on intracellular vesicles, allowing these bacteria to act as a detoxifying organ for the host (Keren *et al.*^2017^). Intracellular vesicles formed by sulfur globules surrounded or not by a proteinaceous membrane have been known to be present in Bacteria for a long time (Bazylinski *et al.*^2004^, ^2013^; Prange *et al.*^2004^). In some bacteria, these vesicles are transient and completely degraded after oxidation of sulfur to sulfate (Franz *et al.*^2007^). In others, they are released into the extracellular medium to avoid a toxic accumulation of sulfur (Eichinger *et al.*^2014^).

The best characterized bacterial intracellular vesicles have been observed in Planctomycetes. These bacteria form a distinct phylum and possess unusual features such as intracellular compartmentalization and proteinaceous cell walls (Fuerst and Sagulenko 2011; Devos and Ward 2014). Intriguingly, they also contain intracytoplasmic membranes which separate cells into multiple functionally distinct compartments (van Niftrik *et al.*^2004^; Gottshall *et al.*^2014^; Sagulenko *et al.*^2017^). In cells of the genus *Gemmata*, invagination of the cytoplasmic membrane forms a complex system of internal membranes (Lindsay *et al.*^2001^) with a network of interconnected vesicles (Acehan, Santarella-Mellwig and Devos 2014). Additionally, the ability of *Gemmata obscuriglobus* to internalize proteins from the extracellular environment may be reminiscent of eukaryotic endocytosis (Lonhienne *et al.*^2010^; Fuerst and Sagulenko 2011). The mechanisms by which these vesicles and membrane invaginations are formed remain unknown; however, several homologs of eukaryotic membrane coat proteins have been detected in the genomes of Planctomycetales (Santarella-Mellwig *et al.*^2010^). Despite the apparent versatility of the cell membranes of Planctomycetes, the production of OMVs by these species has not yet been reported.

### EVs in Eukaryotes: microvesicles, exosomes and apoptotic bodies

Eukaryotes are composed of complex cells characterized by an extensive intracellular network of tubular membranes producing intracellular vesicles, some of these membranes being specialized in particular function (e.g. the nuclear membrane). This network is connected to a cytoplasmic membrane usually rich in glycoproteins and sometimes covered by a thick cell wall (e.g. in plants or fungi). Notably, the basic molecular mechanisms of Eukaryotes are often very divergent from those of Bacteria (sometimes even non-homologous e.g. DNA replication), showing much greater similarity to archaeal systems (e.g, translation, transcription and so on). This has triggered intense debates about the relationships between Archaea and Eukaryotes, with some authors suggesting that Eukaryotes originated from Archaea, whereas others, analyzing the same data, concluded that Eukaryotes and Archaea are two monophyletic groups that share a specific common ancestor (for recent data and discussions on this topic, see Spang *et al*. 2015, 2018; Da Cunha *et al*. 2017, 2018). Beside their archaeal-like component, all eukaryotes also share a strong bacterial heritage since they all contain mitochondria (or relics of them) that were acquired from an intracellular Alphaproteobacteria. Consequently, they exhibit a unique combination of archaeal and bacterial features associated with unique eukaryotic features, such as split genes and the spliceosomal machinery.

The release of EVs to the extracellular space is probably conserved in all types of eukaryotic cells: animals, plants, protists and fungi, be they either in unicellular or multicellular organisms; however, most studies to date have been done in animals, specifically in the two mammalian models, mouse and human. EVs produced by human cells have been studied for quite a long time now (see Yáñez-Mó *et al.*^2015^; Stahl and Raposo 2018; Tkach, Kowal and Théry 2018 for brief histories). These EVs can be found in diverse biological fluids from amniotic fluid to urine, breast milk, saliva and even cerebrospinal fluid (Mathivanan and Simpson 2009; Kalra *et al.*^2012^; Kim *et al*. 2013, 2015; Yáñez-Mó *et al.*^2015^; Kalra, Drummen and Mathivanan 2016; Maas, Breakefield and Weaver 2017; De la Torre Gomez *et al.*^2018^; Stahl and Raposo 2018; van Niel, D’Angelo and Raposo 2018). They are also an important component of the extracellular matrix (Rilla *et al.*^2017^). Notably, EVs produced by eukaryotic cells have the ability to deliver their cargoes not only to neighboring cells in their tissue microenvironment, but also at long distances throughout the body of multicellular organisms. In particular, they can trigger epigenetic reprogramming by delivering active RNA or DNA to recipient cells.

In humans, increasing evidence suggests that EVs play a fundamental biological role in the regulation of normal physiological and disease processes (Gatti *et al.*^2011^; Raposo and Stoorvogel 2013; Kowal, Tkach and Théry 2014; Yáñez-Mó *et al.*^2015^; Maas, Breakefield and Weaver 2017; Rilla *et al.*^2017^). In cancerous cells, the release of EVs is greatly enhanced and the composition of vesicular proteins, mRNAs and miRNAs varies significantly from healthy cells (Inal *et al.*^2013^; Ohno, Ishikawa and Kuroda 2013; Kreger *et al.*^2016^; Takahashi *et al.*^2017^; Xu *et al.*^2018^). EVs are thus believed to play an important role in tumor proliferation and evading the immune system (Al-Nedawi *et al.*^2008^; van Doormaal *et al.*^2009^; Muralidharan-Chari *et al.*^2010^; Lee *et al.*^2011^; Tricarico, Clancy and D'Souza-Schorey 2016; Whiteside 2016; Naito *et al.*^2017^; Weidle *et al.*^2017^; Xu *et al.*^2018^) and there is a great deal of interest in harnessing EVs as potential biomarkers for the diagnosis and monitoring of cancer (Muralidharan-Chari *et al.*^2010^; Verma *et al.*^2015^; Kinoshita *et al.*^2017^; Chen *et al.*^2018^; Xu *et al.*^2018^). Additionally, EVs have been implicated in spreading neuropathological diseases through the brain via the transport of amyloid proteins (Coleman and Hill 2015) or prions (Hartmann *et al.*^2017^; Liu *et al.*^2017^). Finally, EVs also play a role in aging, with even senescent cells effecting telomere regulation and gene expression of other tissues through EV-mediated mechanisms (Acosta *et al.*^2013^; Takasugi 2018).

Eukaryotic EVs are usually classified into three main categories, based on their mode of production in animal cells: microvesicles (50–1000 nm), exosomes (40–100 nm) and apoptotic bodies (800–5000 nm) (for recent reviews, see Kalra, Drummen and Mathivanan 2016; Maas, Breakefield and Weaver 2017; Stahl and Raposo 2018; van Niel, D’Angelo and Raposo 2018). Microvesicles (also sometimes referred to as microparticles or ectosomes) are formed by the outward budding of membrane vesicles from the cell surface (Fig. 4) (Muralidharan-Chari *et al.*^2009^). In some cases, they are released from tubular structures extending from the plasma membrane (Rilla *et al*. 2013, 2014). Microvesicles thus share some properties with EVs produced by some monoderm bacteria and some archaea (see below).

In contrast, exosomes are specific to eukaryotic cells, being formed through the endocytic pathway from the ‘outward’ budding of the late endosomal membrane (see Box 1) (Harding, Heuser and Stahl 1983; Pan and Johnstone 1983). They first accumulate in these endosomes that became known as multivesicular bodies (MVBs). The MVBs can either fuse with lysosomes, leading to the degradation and recycling of contents, or fuse with the plasma membrane and release their contents as exosomes into the extracellular space (Fig. 4). It is not clear what determines their fate for either degradation or fusion with the plasma membrane.

The third major type of eukaryotic EVs called apoptotic bodies is also specific to eukaryotic cells. They are produced during programmed cell death by outward budding from the surface of apoptotic cell (Fig. 4) (van der Pol *et al.*^2012^). They are usually larger than other vesicles, although their size range somewhat overlaps with that of microvesicles. Apoptotic bodies can contain organelles and/or nuclear remnants and are morphologically diverse (Bilyy *et al.*^2012^). They play an important biological role not only in development but also in the pathogenesis of several disease processes.

Additionally, several types of EVs are produced by specific cell types under specific circumstances (Fig. 4): for example, large oncosomes produced by cells from advanced cancers (Minciacchi *et al.*^2015^), migrasomes produced by migrating amoeboid cells (Ma *et al.*^2015^) and giant vesicles produced by breast cancer cells in the presence of estradiol (Wright *et al.*^2014^). Due to cell-specific nature of these EV subtypes, and their relatively small bodies of literature, our review will not discuss these cases.

**Box 1.**

The term exosome, which can from ‘membrane exfoliated vesicles’, has a confusing history since it was used for the first time to name microvesicles released by different cultured cells and carrying a 5΄-nucleotidase activity (Trams *et al.*^1981^). However, in the early 1980s, a more complex EV secretion pathway, in which intraluminal vesicles formed within MVBs, was described (Harding, Heuser and Stahl 1983; Pan and Johnstone 1983). The existence of this secretion pathway was later also confirmed in antigen-presenting cells, epithelial and tumor cells (Raposo *et al.*^1996^; van Niel *et al.*^2001^; Wolfers *et al.*^2001^). From 1987 onwards, the term exosome was adopted to refer to EVs of endosomal origin (Johnstone *et al.*^1987^). For early publications on diverse eukaryotic EVs, see for instance Kerr, Wyllie and Currie (1972); Friend *et al.* (1978); Raposo *et al.* (1996); Heijnen *et al.* (1999), Théry *et al.* (2001); Hristov *et al.* (2004); Del Conde *et al.* (2005) and Ratajczak *et al.* (2006).

EVs present in circulating fluids are likely to be mainly composed of both exosomes and microvesicles (Muralidharan-Chari *et al.*^2010^). Several studies have shown that EV populations are usually heterogeneous, even in pure cell culture, with each type of cell being able to produce different types of EVs. Moreover, it seems that specific types of EVs (or at least EVs with specific cargoes) may be produced exclusively by different cell types. For example, proteomic analysis of EVs purified from breast milk showed that 198 of the identified proteins are not present in the EV database, Vesiclepedia, suggesting that milk-derived EVs harbor proteins not observed in other EVs (van Herwijnen *et al.*^2016^). This combination of specific EVs being produced by specific cell types and multiple EV subtypes being produced by each cell highlights not only the heterogeneity in EVs, but also the difficulty in studying these enigmatic entities.

One of the major challenges today is to define methods that allow discrimination between these species of vesicles. Current methods of isolation and purification include ultracentrifugation, density gradient centrifugation, affinity chromatography, immuno-affinity methods (Mathivanan *et al.*^2010^; Gardiner *et al.*^2016^; Kowal *et al*. 2016, 2017), ligands reactive with EV surfaces (e.g. heparin) (Atai *et al.*^2013^), separation by charge (Graner *et al.*^2009^; Deregibus *et al.*^2016^) or size by field-flow fractionation techniques (Sitar *et al.*^2015^; Zhang and Lyden 2018) and polymer-based precipitation (Brown and Yin 2017). Exosomes and microvesicles are considered molecularly different in practice due to their different modes of production; however, it can be difficult to distinguish between them; thus, most purification techniques isolate mixed EV populations. Microvesicles (50–1000 nm) are typically larger and more heterogeneous than exosomes (40–100 nm) but their size range does overlap. Exosomes are enriched in the fraction of small EVs with diameter less than 200 nm, but this fraction does also contain microvesicles. As a consequence, EVs isolated by ultracentrifugation are likely to contain a mixed population of both. This has been clearly demonstrated by Théry and colleagues who isolated four different types of EVs from human primary dendritic cells by a combination of ultracentrifugation and density gradient centrifugation (Kowal *et al.*^2016^). They proposed the use of immunoisolation using exosome/microvesicle-specific antigens. To complicate matters further, the content of EVs varies depending on the source and original isolation or enrichment techniques. Thus, care must be taken before assigning functions to one EV type that could be due to other EVs present in the preparation (Théry *et al.*^2006^; Tkach and Théry 2016), and many authors now suggest calling vesicles sedimenting at 100 000 *g* as ‘small EVs’ rather than exosomes (Mateescu *et al.*^2017^).

In an attempt to identify the content of EV preparations, several proteins have been proposed as markers of exosomes including major histocompatibility complex, tetraspanins, ALIX proteins, flotillin, TSG101, heat-shock proteins or Rab5b (Chen *et al.*^2015^; see table 1 in Tkach and Théry 2016 and references therein). A position paper by ISEV suggested that studies should demonstrate a minimum of three of these marker proteins in EV preparations to confirm the presence of exosomes (Lötvall *et al.*^2014^). However, subsequent studies showed that even these markers are not exclusive to exosomes (Kowal *et al.*^2016^). An international consortium of EV scientists recently set up the EV-TRACK knowledge base (http://evtrack.org) to collect and normalize the various methodologies used to isolate EVs in the hope of increasing transparency and reproducibility (van Deun *et al.*^2017^; Witwer *et al.*^2017^).

### Cargo of eukaryotic EVs

Both exosomes and microvesicles are formed through the packaging of cytoplasmic contents in membrane-bound vesicles, and thus have been shown to carry all types of cellular components: proteins, lipids, carbohydrates, DNA and RNAs (mRNA, microRNA and other non-coding RNAs) (El Andaloussi *et al.*^2013^; Penfornis *et al.*^2016^, Mateescu *et al.*^2017^; Record *et al.*^2018^). Several proteins are enriched in EVs, and these may provide clues as to the biogenesis and/or physiological roles of EVs. Enriched proteins include lipid raft-interacting proteins, tetraspanins and associated proteins; immunoglobulins and growth factor receptors; cytoskeletal proteins such as tubulin and actin; ESCRT-related proteins; heat-shock proteins; and proteins involved in vesicle trafficking such as Rab GTPase proteins, annexins and protein of the SPFH (stomatin, prohibitin, flotillin and HflK/C) superfamily, especially stomatin (Snyers, Umlauf and Prohaska 1999; Salzer, Mairhofer and Prohaska 2007; Lapatsina *et al.*^2012^; for a review on proteomic studies of exosomes and microvesicles, refer to Greening *et al.*^2017^). Interestingly, stomatin is known to be a major protein in vesicular lipid rafts, and despite being first detected in Eukaryotes, was later identified in both Bacteria and Archaea, suggesting some degree of conserved membrane dynamics across the three domains (Tavernarakis, Driscoll and Kyrpides 1999; Lee *et al.*^2017^). For an exhaustive review on cargo selection in eukaryotic EVs, see Villarroya-Beltri *et al.* (2014).

Despite being a major component of EVs, lipids have largely been sidelined until recently. Lipidomic studies of EVs from different cell types are required to elucidate the role of lipids in the biogenesis and biological functions of EVs. EVs are enriched in lipids, including sphingomyelin, cholesterol, ganglioside GM3, disaturated lipids, phosphatidylserine and ceramide (Subra *et al.*^2007^; Llorente *et al.*^2013^; Record *et al.*^2014^, ^2018^; Skotland, Sandvig and Llorente 2017). In contrast, the levels of phosphatidylcholine and diacyl-glycerol are decreased relative to the cell (Laulagnier *et al.*^2004^; Skotland, Sandvig and Llorente 2017, and references therein).

Additionally, some differences in lipid composition between exosomes and microvesicles have been observed, and are likely reflective of their different method of biogenesis, either originating from MVBs or the plasma membrane (Bicalho, Holovati and Acker 2013; Zaborowski *et al.*^2015^; Abels and Breakefield 2016). Indeed, these differences in lipid composition and protein-to-lipid ratios of EVs have been suggested to be a more reliable way to characterize EV subpopulations than protein content alone (Osteikoetxea *et al.*^2015^).

Not only are lipids implicated in the biogenesis of vesicles, they also play an important role in EV uptake by cells via lipid raft-mediated internalization (Mulcahy, Pink and Carter 2014). EV uptake is reduced when EV-producing cells are pre-treated with compounds which prevent the biosynthesis of glycosphingolipids. Additionally, sphingolipids of EVs have been shown to have an important role in binding and endocytosis by recipient cells, possibly through cholesterol-rich microdomains (Izquierdo-Useros *et al.*^2009^; Mulcahy, Pink and Carter 2014). Thus, it is clear that both the lipid content of vesicular membranes and the protein cargo which they contain are important at every step of EV function from biogenesis to uptake.

### RNA in EVs

In 2007, Lötvall and colleagues demonstrated that eukaryotic EVs (described as exosomes) contain large amount of RNA, including mRNA and sRNA, and can transfer this RNA to recipient cells (Valadi *et al.*^2007^). Importantly, they reported that mouse mRNA transferred via EVs to recipient human cells could be translated into corresponding mouse proteins. This observation strongly stimulated interest for EVs among cell biologists, especially when it was demonstrated that the transferred RNA can be active in the recipient cell, modifying its phenotype (Skog *et al.*^2008^; Kosaka *et al.*^2010^; Pegtel *et al.*^2010^; Zhang *et al.*^2010^). Since that time, a number of studies have confirmed these preliminary observations (for reviews, see Abels and Breakfield 2016; Mateescu *et al.*^2017^).

Eukaryotic EVs from animals, plants, fungi and protists contain an abundance of different types of potentially active RNA, such as mRNAs, miRNAs and rRNAs, long and short non-coding RNA, tRNA fragments, piwi-interacting RNA, vault RNA and Y RNA as well as RNA-binding proteins that are probably involved in RNA selection and delivery (see below). Many of these RNAs are selectively enriched or depleted in EVs relative to their host cells, and even between different EV subpopulations, suggesting an active mechanism of RNA packaging (Abels and Breakefield 2016; Wei *et al.*^2017^). Although there is some intact mRNA and long non-coding RNAs present in EVs, most of the RNA is fragmented or of small size (Batagov and Kurochkin 2013; Wei *et al.*^2017^). As mentioned above, these vesicle-encapsulated RNAs can have a profound impact on recipient cells, transferring between different cell types causing transient transformation of recipient cells e.g. resulting in production of novel proteins (in the case of mRNA transfer), or regulation of gene expression (in the case of miRNAs) (Valadi *et al.*^2007^; Skog *et al.*^2008^; van der Vos *et al.*^2016^). This process has gained significant attention in the field of cancer research, where it has been observed that tumor-derived EVs can promote tumorigenesis in healthy cells and prime tissues to become future metastatic sites through the transfer of RNAs (Peinado *et al.*^2012^; Zomer *et al.*^2015^). Despite the importance of EV-derived RNAs, this is a field fraught with technical challenges—combining the issues of EV purification and identification (discussed above) with the delicate nature of minute RNA samples. As such, few standards are available to compare studies across various fields (Mateescu *et al.*^2017^).

Interesting results have already been obtained on the mechanisms of miRNA sorting into eukaryotic EVs. Understanding these mechanisms could eventually make it possible to selectively modify RNA cargoes for therapeutic purposes. Recognition of specific RNA nucleotide sequences motifs by exosomal RNA binding proteins is implicated in miRNA sorting. These motifs were detected in miRNA enriched in exosomes using bioinformatic (Batagov, Kuznetsov and Kurochkin 2011; Villarroya-Beltri *et al.*^2013^) or biochemical approaches (Santangelo, Giurato and Cicchini 2016). Bagatov and colleagues identified three 8 nucleotides motives enriched in exosomal RNA sequences extracted from gene expression databases. Later on, Kossinova and colleagues succeeded in isolating two human RNA-binding proteins present in exosomes (YB-1 and NSUN2) that bind short RNA hairpins containing these motifs (Kossinova *et al.*^2017^). Sanchez-Mardrid and colleagues identified GGAG as a motif enriched into miRNA of exosomes produced by human lymphoblasts (Villarroya-Beltri *et al.*^2013^). They found that an RNA-binding protein (hnRBP2B1) specifically binds miRNA through the recognition of this motif and controls their loading into exosomes. This mechanism appears to be itself controlled by sumoylation of this protein that triggers its binding to miRNA. More recently, using miRNA enriched in exosomes as baits, Santangelo and colleagues isolated from human hepatocyte cells another RNA-binding protein (SYNCRIP/hnRNP-Q) that specifically binds to miRNA containing the motif GGCU at their 3΄ end (Santangelo, Giurato and Cicchini 2016). SYNCRIP knockout prevents sorting of these miRNAs in exosomes. Remarkably, introducing a sequence (hEXO motif) containing the GGCU motif in an miRNA normally absent in exosome promoted its exosomal export. All these experiments confirm that RNA packaging in EVs is not a random but a highly regulated process, suggesting that the same should be true for all other types of cargoes present in EVs.

### DNA in eukaryotic EVs

Relative to EV-RNA, less is known about the DNA content of eukaryotic EVs. There are reports of single-stranded DNA (Balaj *et al.*^2011^), mitochondrial DNA (Guescini *et al.*^2010^), plasmid DNA (Shader 2014) and double-stranded DNA (Thakur *et al.*^2014^); for recent reviews, see Cai *et al*. (2013, 2016). DNA appears present as small fragments (around 10–20 kb) and appears to be randomly selected from the entire genome, including mitochondrial DNA. Importantly, some DNA fragments associated with EVs contain entire genes with promoter and terminator regions. This DNA can be transported from cell to cell by endocytosis or fusion and this transfer can affect the transcription pattern of the recipient cells, inducing both upregulation and downregulation of many genes (Waldenström *et al.*^2012^). EV-mediated transfer of DNA coding for mRNA transcripts can thus affect cellular functions and could play an important role in the progression of various diseases (see many examples in the review by Cai *et al.*^2016^). Many aspects of this mechanism remained to be clarified. Notably, many studies have failed to conclusively demonstrate whether the nucleic acids are within or associated with the surface of EVs. Furthermore, whereas the presence of DNA in apoptotic bodies is easy to understand, the mechanism of DNA packaging in exosomes or microvesicles remains unclear.

### Biogenesis of exosomes

The biogenesis of exosomes is a complex process that involves two main steps: their blebbing from the late endosome membrane leading to their accumulation in the MVB and the fusion of the MVB with the cytoplasmic membrane to release exosomes in the extracellular space (for a review, see Abels and Breakefield 2016, and references therein).

The role of the ESCRT (endosomal sorting complex required for transport) machinery in the first step is widely accepted. This machinery comprises four complexes (0, I, II and III) and many associated proteins (such as VPS4, VTA1, ALIX, and TSG101). Two ESCRT-dependent pathways for exosome biosynthesis have been described, with much research having been done to elucidate the mechanisms involved (Abels and Breakefield 2016).

In addition, ESCRT-independent pathways are likely be involved in exosome formation, though these are less well understood. Indeed, the depletion of key proteins in different ESCRT complexes does not abolish MVB formation (Trajkovic *et al.*^2008^; Stuffers *et al.*^2009^). These ESCRT-independent mechanisms are thought to involve lipids, tetraspanins or heat shock proteins.

Many enzymes involved in the modification of membrane lipids (such as phospholipase D) have been shown to regulate exosome secretion (Laulagnier *et al.*^2004^; Trajkovic *et al.*^2008^; Babst 2011; Record *et al.*^2018^). These lipids appear to become concentrated into endosomal regions of the membrane causing deformation and initiating vesicle production—a generalized mechanism similar to that found in some bacteria (see above).

As mentioned earlier, ESCRT proteins and other proteins involved in exosome biogenesis, such as tetraspanins, and ALIX, have been identified from purified exosomes in several proteomic studies and used as markers to distinguish exosomes from other types of EVs (reviewed in Choi *et al.*2015b). However, it is becoming apparent that the diversity of mechanisms of exosome formation parallels the diversity of exosome themselves. Furthermore, considering the difficulty to separate exosomes from microvesicles, and the heterogeneity of exosome, it is sometimes unclear if all reported mechanisms are really specific for exosomes.

The second step in the formation of exosomes, their release into the extracellular space, involves the fusion of the MVB endosomal membrane with the plasma membrane (Abels and Breakefield 2016). As with many other membrane fusion processes in Eukaryotes, the SNARE proteins (and proteins which modify their activity) appear to play an important role in exosome release (Gross *et al.*^2012^; Ruiz-Martinez *et al.*^2016^; Wei *et al.*^2017^).

Additionally, many studies have identified the small GTPases of the Rab and Ras families as playing a role in the regulation of exosome secretion (Hsu *et al.*^2010^; Ostrowski *et al.*^2010^; Koles *et al.*^2012^; for a review, see Hessvik and Llorente 2018). These proteins are membrane anchored and are thought to promote fusion of the MVB with the cell membrane. Indeed, cells with mutant forms of these proteins release fewer exosomes than their wild-type counterparts (Ostrowski *et al.*^2010^).

### Biogenesis of microvesicles

Microvesicle biogenesis is less well understood than that of exosomes, though it is becoming increasingly evident that these processes share some of the same machinery. Microvesicles released from cancer cells were shown to be pinched from the membrane via actomyosin-based contraction, mediated in-part by the small Ras-related GTPase, ARF6 (Muralidharan-Chari *et al.*^2009^). ARF6 is known to activate phospholipase D in a pathway which results microvesicle budding (Muralidharan-Chari *et al.*^2009^; Tricarico, Clancy and D'Souza-Schorey 2016). More recently, Rab-family proteins have also been shown to co-localize to microvesicles and an overexpression of Rab22a led to increased levels of microvesicle budding (Wang *et al.*^2014^; Tricarico, Clancy and D'Souza-Schorey 2016).

In *Caenorhabditis elegans*, ESCRT-I is required for early stages of microvesicle formation (Wehman *et al.*^2011^), whereas ESCRT-II and ESCRT-III subunits are dispensable for microvesicle biogenesis (Wehman *et al.*^2011^). Additionally, in humans ESCRT-I is recruited for microvesicle release from the plasma membrane (Nabhan *et al.*^2012^) supporting a connection between the ESCRT pathway and microvesicle formation.

EV release has been shown to take place at specific locations on the cell membrane that are enriched in assorted lipids and proteins. Lipids with similar shape cluster together forming a monolayer that adopts the spontaneous curvature of the local lipids (McMahon and Gallop 2005; Cooke and Deserno 2006). Lipid bilayers in eukaryotic cell membranes employ active mechanisms to resist spontaneous curvature, and these have been conserved throughout evolution. Thus, the likelihood of spontaneous membrane curvature being the driving force behind *de novo* vesiculation is assumed to be low due to energy constraints. However, membrane curvature could be triggered by active alterations in lipid and/or protein composition. An uneven distribution of lipids between the two membrane leaflets may affect membrane rigidity and curvature and result in budding. It has also been reported that Ca^2^^+^-dependent aminophospholipid translocases (flippase and floppases) contribute to the formation of membrane curvature during microvesicle formation and during formation and fission of Golgi vesicles *in vitro* (D’Souza-Schorey and Clancy 2012; Tricarico, Clancy and D'Souza-Schorey 2016; van Niel, D’Angelo and Raposo 2018). Additional components of lipid bilayers have been implicated in vesicle biogenesis. For example, cholesterol—a key component of membrane lipid rafts—could play a role in the ‘pinching’ events (Muralidharan-Chari *et al.*^2010^). Depletion of cholesterol has been shown to impair microvesicle shedding (Del Conde *et al.*^2005^; van Niel, D’Angelo and Raposo 2018). On the other hand, proteins may exert a localized normal force, pushing on the membrane to generate the curvature needed to begin the budding process (Boulbitch 1998; Farsad and De Camilli 2003). Proteins can bend membranes by binding to the membrane surface and force curvature as a result of increasing the surface area of one leaflet (Sheetz, Painter and Singer 1976). Protein crowding at the surface of the cell membrane can also generate pressure via protein–protein interactions and drive membrane bending (Stachowiak *et al.*^2012^). A recent study showed that even green fluorescent protein was capable of driving fission when attached to membrane surfaces under crowded conditions (Snead *et al.*^2017^). This raises the possibility that the simple enrichment of protein cargo at sites of microvesicle budding could be sufficient to drive *de novo* microvesicle formation (Tricarico, Clancy and D'Souza-Schorey 2016).

It is interesting to note that the release of microvesicles also resembles the events associated with viral budding (see below) (Chazal and Gerlier 2003; Morita and Sundquist 2004; Meckes and Raab-Traub 2011; Wurdinger *et al.*^2012^) and the release of apoptotic bodies, both of which form by outward protrusion of the plasma membrane. However, unlike apoptotic bodies, microvesicles do not contain fragments of cytosolic organelles (Taylor and Gercel-Taylor 2008; Crescitelli *et al.*^2013^).

### Biogenesis of apoptotic bodies

Apoptotic bodies are produced when cells undergo fragmentation during apoptosis. Their production was originally believed to be a stochastic process; however, recent work has demonstrated that the formation of apoptotic bodies is a highly regulated multistep process (Chekeni *et al.*^2010^; Poon *et al.*^2014^; Atkin-Smith *et al.*^2015^). Interestingly, the group of Poon also observed vesicles resembling ‘beads-on-a-string’ being formed in apoptotic cells. They referred to these string-like structures as apoptopodia (Atkin-Smith *et al.*^2015^).

Curiously a study by the group of Lötvall (Crescitelli *et al.*^2013^) showed that RNAs isolated from apoptotic bodies, membrane vesicles and exosomes have very different profiles. Indeed, they observed that membrane vesicles had the least amount of RNA, with rRNA being primarily found in apoptotic bodies. Perhaps this reflects the more active cargo selection of microvesicles, relative to the packaging of a fragmented cytoplasm (containing many ribosomes) in apoptotic bodies.

### Nanotubes in Eukaryotes

Nanotubes were first described in Eukaryotes as tunneling nanotubes (TNTs) and have been observed to connect cells over long distances and transfer vesicles (Fig. 2a), membrane proteins, cellular components (including mitochondria), prions, miRNA and viruses from cell to cell (Rustom *et al.*^2004^; Davis and Sowinski 2008; Lou *et al.*^2012^; for recent reviews, see Austefjord, Gerdes and Wang 2014; Rustom 2016; Nawaz and Fatima 2017). TNTs formed between animal cells have been likened not only to Plasmodesmata connecting plant cells but also to bacterial nanopods (Fig. 2b) (Rustom 2016). These nanotubes seem to be formed from intracellular vesicles, and their shape and movement are determined by the action of cytoskeletal proteins (Rustom 2016). Most of them contain actin filaments along their entire length, possibly explaining their rigidity. Inhibitors of actin polymerization significantly decrease the number and length of TNTs that occur between trabecular meshwork cells and reduce EV transfer, whereas compounds that stabilize actin filaments increase EV transfer (Keller *et al.*^2017^). Some eukaryotic TNTs also contain tubulin and are usually larger. Eukaryotic nanotubes are indeed variable, with diameters varying from 50 to 700 nm (Austefjord, Gerdes and Wang 2014; Bénard *et al.*^2015^). Bénard *et al.* (2015) have shown that the same cells can produce different types of TNTs that can be discriminated by their size and protein content, with larger TNTs (diameter 100–650 nm) containing both actin and tubulin and smaller ones (70–200 nm) containing only actin. These structures appear larger than archaeal and bacterial nanotubes.

Nanotube-like structures containing actin were also described in some cancer cells as ‘protrusions’ related to filipodia and directly associated with the production of microvesicles. Rilla *et al.* (2013) reported that the tip of these protrusions can detach into the culture medium as microvesicles. The formation of both protrusions and microvesicles was enhanced by overexpression of hyaluronan synthase in human mesothelial cells suggesting active regulation of these structures (Rilla *et al.*^2013^; Koistinen *et al.*^2017^). Despite their similarity, the relationship between these protrusions and TNTs is unclear.

Recently, the effect of vesicle size on nanotube formation *in vitro* and *in vivo* was investigated using lysosomes and autolysosomes (Su *et al.*^2016^). The results suggested that tubulation was dependent on the size of the vesicles in the cell, reinforcing the link between nanotubes and vesicles.

### The ubiquity of EVs in Eukaryotes

The study of EVs in Eukaryotes other than humans (and a few animal models) is lagging far behind the study of human exosomes and microvesicles. However, it is already clear that all eukaryotic cells, from both unicellular and multicellular organisms, produce these two types of EVs.

Plant cells have also been shown to be producers of exosome-like vesicles, especially in response to pathogen attack (Stanly *et al.*^2016^; Rutter and Innes 2017). For instance, *Arabidopsis* can deliver small siRNAs into the fungal pathogen *Botrytis cinecrea* via EVs. These siRNAs silence genes critical for pathogenicity (Cai *et al.*^2018^). Proteomic analysis on EVs purified from apoplastic fluids of *Arabidopsis* leaves revealed that they are highly enriched in biotic and abiotic stress response proteins (Rutter and Innes 2017). Indeed, increased levels of EVs were observed when plants were infected with *P. syringae* or treated with salicylic acid. Thus, EVs may play an important role in the immune responses of plants.

Fungi were hypothesized to release EVs outside their cell membrane as early as the 1970s following observations in *Saccharomyces cerevisiae* and *Cryptococcus neoformans* (Takeo, Uehira and Nishiura 1973; Novick and Schekman 1979). Later, EV production by *Candida albicans* was observed by TEM and scanning electron microscopy (SEM) (Anderson, Mihalik and Soll 1990). For a long time, these observations were not followed up, largely due to the belief that the structure of the fungal cell wall was too rigid to allow budding. However, production of EVs by *C. neoformans* was finally confirmed (Rodrigues *et al.*^2007^) and EV production was subsequently associated with a variety of other fungi (Rodrigues *et al.*^2007^, ^2014^; Albuquerque *et al.*^2008^; Nosanchuk *et al.*^2008^; Oliveira *et al.* 2010a,b ). EVs produced by fungal cells are similar to mammalian exosomes, (Albuquerque *et al.*^2008^; Rodrigues *et al.*^2008^) including their ability to modulate the function of immune cells (Oliveira *et al.* 2010a,b) and export RNA (Peres da Silva *et al.*^2015^; Bielska *et al.*^2018^). Recently, it was shown that EVs produced by the human pathogen *Cryptococcus gattii* can be delivered to intracellular fungal cells within macrophages and trigger their division (Bielska *et al.*^2018^). The RNA and proteins associated with these EVs are essential for this long-distance pathogen-to-pathogen communication.

The EVs are probably excreted from fungal cells through the pores (from 50 to 500 nm) that are present in the thick cell wall. The size and abundance of these pores is constantly remodeled during the cell cycle, possibly controlling the release of EVs. In agreement with the hypothesis that EV release is an active process, EVs in fungi are produced exclusively from living cells. Finally, EVs from fungi contain β-glucosidase and endochitinase that can help to reduce the thickness of the wall to facilitate EV crossing (Brown *et al.*^2015^ and references therein).

A broad range of eukaryotic microbes, such as the ‘algae’ *Emiliania huxleyi*, the Amoeobozoa *Dictyostelium discoideum* and *Acanthamoeba*, including several extracellular parasites (e.g. *Trichomonas vaginalis*, *Trypanosoma cruzi*, *Leishmania* spp. and helminths) and intracellular parasites (*Plasmodium falciparum*, *Toxoplasma gondii* and *Leishmania* spp.), have been shown to produce EVs (Bhatnagar *et al.*^2007^; Silverman *et al.*^2008^, ^2010^; Martin-Jaular *et al.*^2011^; Cestari *et al.*^2012^; Marcilla *et al.*^2012^, ^2014^; Mantel *et al.*^2013^; Pope and Lasser 2013; Regev-Rudzki *et al.*^2013^; Twu *et al.*^2013^; Arantes *et al.*^2016^; Marti and Johnson 2016; Schatz *et al.*^2017^; Ribeiro *et al.*^2018^). These EVs can play a major role in the interactions between the parasites and their host; for example, *Leishmania* spp. have been shown to constitutively secrete exosomes within the lumen of the sand fly midgut and, following their ingestion into a new host during the insect's bite, these vesicles play an important role in modulation of host immunity, and thus promote the parasitic infection (Atayde *et al.*^2015^). In *T. cruzi*, it has been shown recently that differences in EV production between strains correlate with their infectivity and virulence (Ribeiro *et al.*^2018^). The production of EVs has been also observed in studying the infection of eukaryotic microbes by viruses. The production of EVs by *Acanthamoeba* was dramatically illustrated by the production of large vesicles containing virions from cells infected by the giant virus *Marseillevirus* (Arantes *et al.*^2016^). Additionally, the interplay between viruses and EVs has been nicely illustrated by analyzing EV production during the infection of the *E. huxleyi* by EhV, Phycodnaviridae (Schatz *et al.*^2017^). These are discussed further below.

The Amoebozoa *D. discoideum* has been proposed as a model organism for the study of eukaryotic EVs (Tatischeff 2013). Besides producing EVs implicated in the types of intercellular communication observed in other systems, it was demonstrated that *Dictyostelium* was able to lessen the toxicity of drugs, such as Hoechst 33342 (HO342), by secretion into EVs (Lavialle *et al.*^2009^).

### EVs in Archaea

Archaea are unicellular microorganisms that look like bacteria at the phenotypic level. In particular, they lack nuclei and are often grouped with Bacteria as being prokaryotes. However, their informational systems (transcription, translation, and DNA replication) as well as some mechanisms associated with their membranes (e.g. the Sec secretion system, the ATP synthase complex, the signal recognition particles) are much more similar to those of eukaryotes. Some archaea even contain proteins closely related to eukaryotic actin and tubulins (Ettema, Lindås and Bernander 2011; Yutin and Koonin 2012; Lindås *et al*. 2017) as well as proteins involved in membrane remodeling and eukaryotic EV formation such as ESCRT III proteins and/or VPS4 ATPases (Makarova *et al.*^2010^). All cultured archaea lack peptidoglycan and most of them are monoderm (Ellen *et al.*^2010^; Albers and Meyer 2011), with the exception of *Ignicoccus hospitalis* and Methanomassilicocales which have an OM and a large periplasmic space. In most archaea, the cytoplasmic membrane is surrounded by a crystalline protein S-layer. The S-layer is usually composed of a single main protein type (40–200 kDa) arranged into a highly order structure that is capable of self-assembly, both on cell surfaces and in solution *in vitro* as cell-free S-layer ‘ghosts’ (Kish *et al.*^2016^). Some archaea lack this S-layer, such as Thermoplasmatales, whereas others exhibit an additional peptidoglycan-like structure formed from pseudomurein below the S-layer resulting in positive Gram staining (Methanobacteriales and Methanopyrales) (Steenbakkers *et al.*^2006^; Albers and Meyer 2011; Klingl 2014).

**Figure 7.**
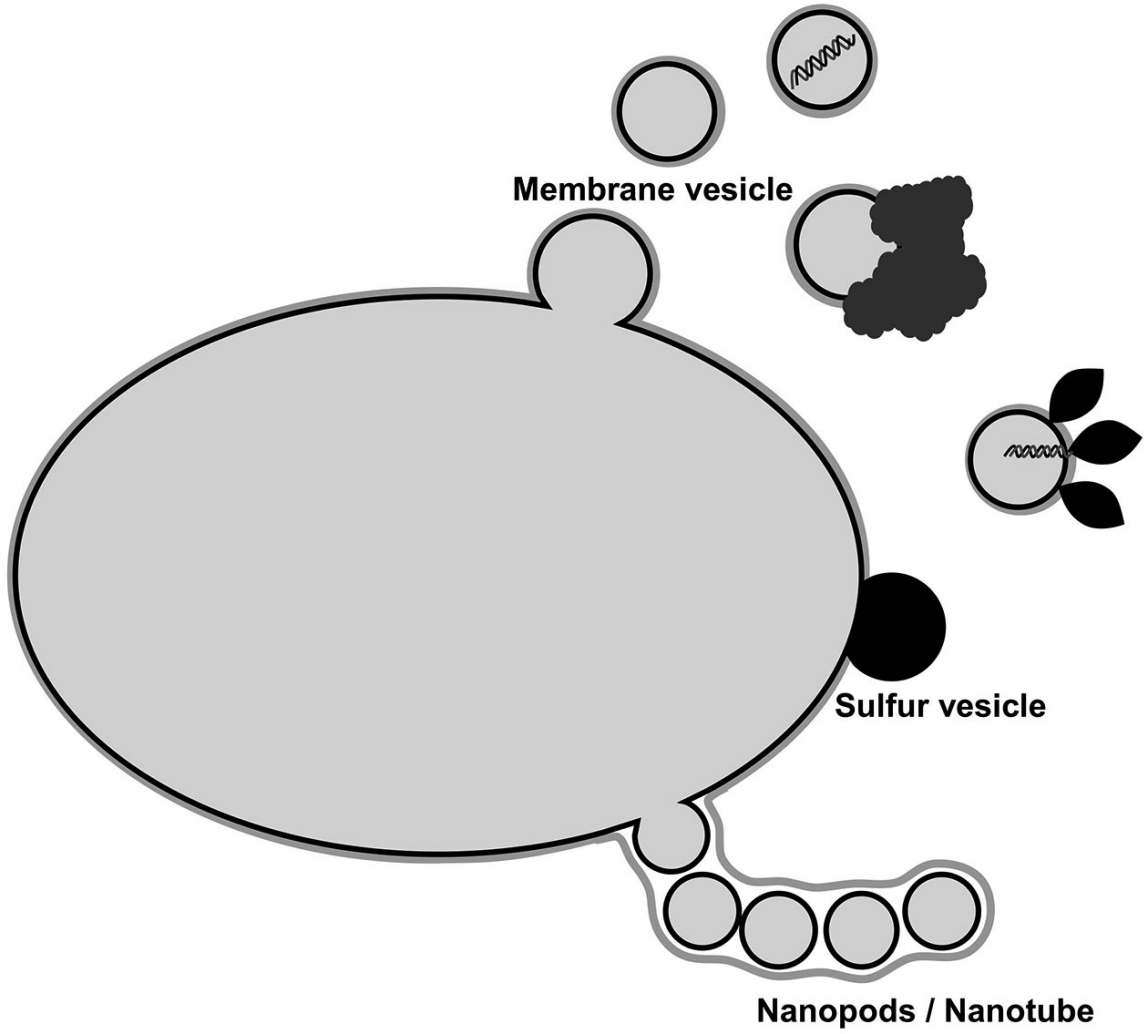
EV production in Archaea. EVs originate from membrane budding and nanotubes. Functions include HGT, intercellular competition, disposal/detoxification, biomineralization and possibly viral defense.

EV production is most likely a general phenomenon in Archaea, as in the other two domains of life (Fig. 7). Archaeal EVs were first observed in the thermoacidophilic archaeon *Sulfolobus islandicus* (Prangishvili *et al.*^2000^), a member of the archaeal phylum Crenarchaeota. *Sulfolobus* EVs (90–230 nm in diameter) are enclosed by cytoplasmic-like membrane and coated with the S-layer. Characterization of EVs produced by three other *Sulfolobus* species, *S. acidocaldarius*, *S. solfataricus* and *S. tokodai*, has shown that the lipid and protein profiles of parental cells membranes and secreted vesicles were different, suggesting a selective mechanism for protein packaging in EVs (Ellen *et al.*^2009^). Interestingly, the proteomic analyses of EVs from the three species revealed the presence of proteins homologous to eukaryotic ESCRT-III subunits and to the VPS4 ATPase. Studies have shown that *Sulfolobus* homologs of ESCRT-III and VPS4 (also called CdvB and CdvC, respectively) are involved in cell division (Lindås *et al.*^2008^; Samson *et al.*^2008^), and it has been suggested that these proteins could be also involved in EV formation by a mechanism similar to that of exosome production in Eukaryotes (Ellen *et al.*^2009^; Caspi and Dekker 2018). In several reviews, authors have indeed assumed that the archaeal homologs of eukaryotic components are involved in EV formation as if it was a well-established result (see for instance figure 4 in Deatherage and Cookson 2012). However, this remains a hypothesis that needs to be proved conclusively using genetic approaches.

Notably, *Sulfolobus* EVs carry an antimicrobial protein, termed ‘sulfolobicin’, that inhibits the growth of related *Sulfolobus* species (Prangishvili *et al.*^2000^). In fact, they were first confused with viral particles because spotting EV preparations on *Sulfolobus* lawns resulted in halos similar to lysis plaques. Later, Driessen and colleagues showed that sulfolobicins are in fact composed of two proteins whose genes are organized in operon (Ellen *et al.*^2011^).

A recent study revealed that *Sulfolobus* EVs could also promote biomineralization (Kish *et al.*^2016^). Whilst S-layers have long been associated with mineral formation, the mechanisms by which this occurs remain unresolved. A study using *S. acidocaldarius*, a hyperthermophilic archaeon native to metal-enriched environments, demonstrated a passive process of iron phosphate nucleation and growth within the S-layer of cells and cell-free S-layer ‘ghosts’ during incubation in a Fe-rich medium. In addition, EVs of ∼ 175 nm in diameter were formed and released as a response to S-layer encrustation by minerals. These vesicles were fully encrusted by minerals, even when cells were only partially encrusted (Kish *et al.*^2016^). The authors propose that these EVs are produced in an attempt to remove sections of damaged S-layer.

Archaeal EVs have been also studied in hyperthermophilic (and neutrophilic) archaea of the genus *Thermococcus* (Fig. 1c and d), which are members of the phylum Euryarchaeota. Extensive screening revealed that most of the strains in this genus released EVs (50–150 nm) that are morphologically very similar (albeit a bit smaller) to those produced by *Sulfolobus* (Soler *et al.*^2008^; Gaudin *et al.*^2013^; Marguet *et al.*^2013^). The major protein present in both the cell membranes and EVs of *Thermococcus* species is the oligopeptide-binding protein OppA (Gaudin *et al.*^2013^), which is also found in *Sulfolobus* EVs (Ellen *et al.*^2009^). However, unlike the situation observed with *Sulfolobus*, the protein profiles of EVs from different species of Thermococcales showed that both EVs and cell membranes from the same species have a similar composition, suggesting that the mode of EV production could be different in *Sulfolobus* and *Thermococcus*. Indeed, homologs of CdvB are missing in *Thermococcus* and other Euryarchaeota (Makarova *et al.*^2010^).

Thin-section analyses by electron microscopy revealed that these EVs from *Thermococcales* are produced by protrusion of the cell membrane along with the S-layer (Figs 1c and d and 7). During these studies, the authors reported for the first time the presence of rows of EVs enclosed within the S-layer (Soler *et al.*^2008^) resembling the aforementioned nanopods or nanotubes later observed in Bacteria (Fig. 2) (Shetty *et al.*^2011^). EVs present within nanotubes are usually smaller than free EVs but large EVs are frequently observed at the extremities of nanopods, suggesting that these structures could be involved in the transport and/or formation of EVs. These nanopods from Thermococcales sometimes connect cells together (Marguet *et al.*^2013^) and could be involved in transfer of material between cells as in the case of their bacterial counterparts. Finally, these authors also observed ‘nanospheres’ budding from the cell surface, in which several EVs are enclosed by S-layer in a spherical structure (Marguet *et al.*^2013^).

EVs produced by *Thermococcus* species are often associated with genomic DNA (Soler *et al.*^2008^, Gaudin *et al.*^2014^; Choi *et al.*2015a) and RNA (Choi *et al.*2015a; Gill and Forterre unpublished observation). The association of DNA with EVs might be significant for hyperthermophilic species since this vesicle-associated DNA appears to be more resistant to thermodegradation than free DNA (Soler *et al.*^2008^). Notably, whereas HGT between hyperthermophilic species (including transfer between Archaea and Bacteria) has been clearly demonstrated *in silico* by comparative genomics, it is unclear how these transfers can occur at temperatures that provoke DNA melting/degradation. It thus makes sense to propose that EVs could play a major role in gene transfer between hyperthermophilic species. Forterre and colleagues demonstrated that EVs can transfer DNA at least between cells of the same species using a genetically tractable strain *Thermococcus kodakaraensis* (Gaudin *et al.*^2013^). After transformation with a reporter plasmid, this strain produces EVs containing plasmids and these EVs can be used to transfer the plasmid into plasmid-free cells. EVs produced by *T. nautili* naturally harbor DNA of the endogenous plasmids pTN1 and pTN3 suggesting a possible role for EVs in plasmid transfer *in vivo*. Interestingly, the plasmid pTN3 appears to be a viral genome, carrying genes encoding the major capsid protein and the packaging ATPases characteristic of the adenovirus/PRD1 lineage. This suggests that EVs may also be involved in the generation of new viral forms (Soler *et al.*^2011^; Gaudin *et al.*^2014^).

EVs have been also characterized in *T. onnurineus* (Choi *et al.*2015a). The authors separated different populations of EVs showing different buoyant densities by density gradient centrifugation. All of the recovered populations were associated with DNA fragments estimated to be around 14 kb long. Surprisingly, sequencing of the associated DNA revealed that all regions of the *T. onnurineus* genome were present except for a 9.4-kb region. The authors then speculated that this region could encode proteins involved in the mechanism of EV production or in EV-mediated DNA transfer. Interestingly, a *T. onnurineus* mutant in which this region has been deleted still produces vesicles but without associated DNA, favoring the second hypothesis (Kim YK, personal communication). This 9.4-Kb region encodes various enzymes involved in sulfur metabolism and/or hydrogen production and their possible role in DNA packaging is unclear.

Another role that these EVs play in Thermococcales is that of detoxification. Cryo-electron microscopy of *T. prieurii* cells revealed the presence of numerous intracellular dark vesicles that bud from the host cells (Gorlas *et al.*^2015^). These dark vesicles are exclusively found in association with intact cells and are never observed in preparations of purified membrane vesicles. Furthermore, the presence of these vesicles is exclusively observed when elemental sulfur is added into the growth medium. Energy-dispersive-X-ray analyses revealed that these dark vesicles are filled with sulfur, and hence they have been termed ‘sulfur vesicles’ (Fig. 2). These dark vesicles were also observed in *T. kodakaraensis* albeit in lower numbers. Curiously they are lacking in *T. nautili* suggesting that Thermococcales species exhibit significant differences in their sulfur metabolic pathways.

Recently, Erdmann *et al.* (2017) described a novel type of EV containing plasmid in Archaea. These EVs, which are produced by the psychrophilic halophilic archaea *Halorubrum lacusprofundi* (member of the phylum Euryarchaeota), contain a plasmid of 50 kb, pR1S1. These EVs can infect a plasmid-free strain and transform this strain into a producer of EVs containing the plasmid pR1S1. Notably, this plasmid encodes several proteins that are present in the EVs themselves. The authors suggested that these proteins could be involved in plasmid packaging inside EVs and in the formation of an apparatus responsible for the transfer of these EVs from cell to cell. Forterre, Da Cunha and Catchpole (2017) proposed that these EVs be called plasmidions (mimicking virions) to distinguish them from canonical plasmid vesicles that do not contain plasmid-encoded proteins.

The production of EVs is most likely a universal process in Archaea as in the other two domains. EVs and structures resembling nanopods have been observed in cultures of the euryarcheon *Aciduliprofundum boonei* (Reysenbach *et al.*^2006^; Reysenbach and Flores 2008). Additionally, large numbers of EVs were observed in the periplasm of the diderm archaeon *I. hospitalis* harboring several cells of the tiny archaeon *Nanoarchaeum equitans* on its surface (Huber *et al.*^2002^; Küper *et al.*^2010^). The volume of the *Ignicoccus* periplasm can be large (20–1000 nm in width) and contains numerous EVs that bud from the inner membrane and fuse with the OM (Näther and Rachel 2004). *Nanoarchaeum equitans* has the smallest known genome size for an archaeon (0.49 Mb) and cannot synthesize many essential components, including lipids (Waters *et al.*^2003^). Recent evidence suggests that these components are delivered from the cytoplasm of *I. hospitalis* to *N. equitans* via vesicles that reach the OM at the position of the symbiont attachment, or through nanotube-like endomembrane connections between the two cytoplasms (Heimerl *et al.*^2017^).

The mechanisms of EV production remain unknown in Archaea. The lack of simple screening methods for the detection of hypo- and hypervesiculation mutants is a major obstacle. It is likely that different types of EV production mechanisms exist in this domain as in the two others, depending on the species and/or the vesicle types. By analogy with the modes of EV production known in Bacteria and Eukarya, it is possible that EV production in Archaea is controlled by the regulation of enzymes involved in the incorporation of polar lipids in order to create asymmetry between the outer and inner membrane leaflet. However, since many archaea have monolayer cytoplasmic membranes formed by giant tetraether bipolar lipids (e.g. caldarchaeol), classical models of membrane split and fusion which involve the transient opening of the bilayer cannot be applied (Relini *et al.*^1996^).

Monolayer membranes seem to be especially prevalent in hyperthermophilic archaea in which EV production has been widely studied, such as Sulfolobales and Thermococcales. It has been shown that fusion of artificial vesicles made of *Sulfolobus* tetraether lipids is still possible, but is much more difficult than fusion of classical bilayer vesicles (Relini *et al.*^1996^). The authors of this study have shown that fusion is only possible if the lipid vesicles contain monosubstituted molecules in which one of the glycerol head groups is not substituted, and requires trace diether lipids. Importantly, the ratio between tetraether (caldarchaeol) and diether lipids in archaeal membranes can vary depending on the physiological conditions (Meador *et al.*^2014^; Cario *et al.*^2015^ and references therein); thus, it would be interesting to determine if there is a correlation between EV production and lipid composition. In the case of archaea with a predominant monolayer membrane, a hypothetical model could be that membrane curvature is induced by the accumulation of larger polar head groups at the outer surface of the monolayer. Another non-exclusive possibility is that patches of membranes adopt bilayer or simple monolayer structure by favoring diether lipids (archaeol) or horseshoe structures of tetraether, as recently observed in the membrane of the archaeal virus (Kasson *et al.*^2017^).

### EVs and viruses

The size and morphology of EVs are strongly reminiscent of the morphology of some viral particles (virions). A virion (the hallmark of viruses, see Krupovič and Bamford 2010) contains at least one protein encoded by the viral genome. For instance, exosomes strikingly resemble virions of retroviruses and other enveloped viruses (Izquierdo-Useros *et al.*^2011^). Similarly, the virions of Plasmaviridae infecting Mycoplasma, or Pleolipoviridae infecting halophilic archaea, resemble EVs, being formed by a lipid envelope containing inserted viral-encoded proteins (Demina *et al.*^2016^; Erdmann *et al.*^2017^). Some EVs contain viral DNA (viral vesicles) or plasmids (plasmid vesicles and plasmidions) (Fig. 3). These similarities between EVs and viral particles raises a number of interesting questions relative to their respective origins, possible common production mechanisms and possibilities for physiological interactions that we will explore here. They also raise some challenging questions and problems in terms of nomenclature and discrimination between virions and various types of EVs in the environment.

Physical interactions between EVs and virions have been observed in the three domains of life. In Bacteria, Manning and Kuehn (2011) first showed that co-incubation of T4 virions and OMVs showed fast and irreversible binding of these virions to OMVs. Later, Biller and co-workers observed interactions between virions and OMVs in the case of *Prochlorococcus* and the cyanophage PHM-1. They further demonstrated that many vesicle-attached virions had a shortened stalk and altered capsid staining density, suggesting that they had injected their DNA into the vesicle (Biller *et al.*^2014^). More recently, Camilli and colleagues observed by cryo-electron tomography direct attachment of virions from diverse viruses infecting *Vibrio cholerae* to OMVs produced by this bacterium (Reyes-Robles *et al.*^2018^).

All these observations suggest that EVs could be used as antiviral systems by cells, acting as decoys to reduce infectious virus–cell interactions. The efficiency of T4 infection was indeed significantly reduced by the formation of complexes with OMVs (Manning and Kuehn 2011). The same result was obtained with *V. cholerae* infected by the bacteriophage ICP1 (Reyes-Robles *et al.*^2018^). If EVs are used as traps by cells to protect themselves against infection, one expects that cells increase EV production as a response to infection. This is indeed the case: as early as 1978, Loeb and colleagues demonstrated a dramatic increase in OMV production from *E. coli* in the presence of T4 virus (Loeb and Kilner 1978). It has now been reported in several eukaryotic systems that viral infection can increase EV production (Nolte-‘t Hoen *et al.*^2016^). However, in that case, the EVs produced did not act as decoys inhibiting viral infection, but more like Trojan horses favoring infection, facilitating viral infections by delivering viral proteins, RNA and/or DNA from infected to non-infected cells (Altan-Bonnet 2016) (see below).

Direct interactions between viral particles and EVs have been also observed in Archaea, especially between EVs and the lemon-shaped particles produced by *Fuselloviridae* (Geslin *et al.*^2003^) (Fig. 3). However, it is not known whether EVs can interfere with viral infection and how this may occur. Interestingly, the peptide-binding protein OppA detected in EVs of *Sulfolobus* and *Thermococcus* species has been identified as a putative receptor for the *Sulfolobus* virus ATV (Erdmann, Scheele and Garrett 2011). It would be interesting to test if EVs are generally enriched in viral receptors thus promoting virion–vesicle interactions.

Recently, Schatz *et al.* (2017) observed an intriguing interaction between EVs and virions in Eukaryotes when studying the infection of *E. huxleyi* by a phycodnavirus. Notably, this interaction favors the infection process. They show that mixing EVs produced by infected cells with virion preparations prolonged the half-life of the virion from 10 to 60 h. This could increase the chance of infectious encounters between virions and their cellular targets. They proposed that ‘EVs are exploited by viruses to sustain efficient virus infectivity and propagation across *E. huxleyi bloom*s’ (Schatz *et al.*^2017^) with important environmental consequences.

Several studies have shown that EVs produced by virus-infected cells can favor viral infection by making other cells in the population more sensitive to viral infection (similar to that described for cancer metastasis above). For instance, OMVs can transfer viral receptors from sensitive to resistant cells, helping the virus to bypass bacterial resistance due to the lack of receptors in target cells (Tzipilevich, Habusha and Ben-Yehuda 2017). Exchange of phage receptor recognition proteins could even occur between different species, enabling phage adsorption to non-host species, providing an unexplored route for HGT. The fact that viral-infected cells produce EVs that favor viral infection can be understood in the framework of the virocell concept (Forterre 2013), which posits that the infected cell is no more a bacterial, an archaeal or a eukaryotic cell, but a cellular form of the virus (a virocell) whose metabolism and physiology has been remodeled by the virus to fulfill its own objectives. In that view, EVs produced by virus-infected cells can be considered to be viral EVs produced by the virocell, even if they do not contain bona fide viral components.

In many cases, EVs produced by virocells have modified biochemical composition, containing various viral elements—either proteins or nucleic acids. In particular, human cells infected by different types of viruses contain viral proteins, segments of viral RNA, microRNA or mRNA (Pegtel *et al.*^2010^; Meckes and Raab-Traub 2011; Altan-Bonnet 2016; Kouwaki *et al.*^2017^). They can also carry and transport cellular components that make recipient cells more susceptible to viral infection. These viral EVs can modulate the innate immune response system of the host and facilitate viral infection by carrying molecules implicated in virus binding and entry (György *et al.*^2011^; Kouwaki *et al.*^2017^).

In a previously mentioned study, Vardi and colleagues have shown that infection of *E. huxleyi* by its virus (EhV, Physodnaviridae) induces the production of specific EVs (Schatz *et al.*^2017^). These EVs have a unique lipid composition, being strongly enriched in triacylglycerols. Remarkably, the number of EVs produced by virus-infected cell was similar to the number of bona fide viral particles. These EVs can be internalized by virus-free cells, making them more sensitive to viral infection. Cells infected by these EVs are lysed faster than control cells and produce more viral particles. The authors observed modification of cellular sphingolipid metabolism and cell-cycle pathways of ‘EV-infected cells’, probably via a regulatory effect of the EVs sRNA. Notably, the production of EVs can be also triggered by heat-sensitive virus-free lysate from virus-infected cells, indicating that the virus produces one or more proteins that play a direct role in EV production. Addition of this virus-free lysate also increases the formation of triacylglycerols in control cells, again illustrating the link between cellular lipid composition and EV formation. This model system could become especially interesting to decipher the mechanism of EV production in Alga.

EVs containing viral DNA have been called viral vesicles when, unlike virions, they do not contain proteins encoded by the virus (Gaudin *et al.*^2014^). The production of such viral vesicles has been observed in the three domains of life. This definition could even be extended to eukaryotic vesicles containing viral RNA, such as exosomes containing full-length RNA from hepatitis C viruses (Bukong *et al.*^2014^; Longatti, Boyd and Chisari 2015). It has been suggested that viral vesicles could help in the dissemination of viruses in the absence of viral receptors by their capacity to transfer DNA from cell to cell (Soler *et al.*^2015^). The production of viral vesicles has been observed in the three domains of life. Viral vesicles containing genomes from Caudovirales have been described in Bacteria (Yaron *et al.*^2000^), whereas viral vesicles containing a plasmid (pTN3) corresponding to a defective viral genome and plasmid vesicles (or plasmidions) containing infectious plasmids have been described more recently in Archaea (Gaudin *et al.*^2013^; Erdmann *et al.*^2017^). Notably, *in silico* analysis of DNA associated with EVs isolated from diverse marine environments has suggested that viral vesicles could be abundant in nature in addition to true virions and EVs containing cellular DNA (Soler *et al.*^2015^). Further work is now required to determine if viral vesicles and plasmidions indeed play an important role in the co-evolution of viruses, plasmids and their hosts in natural environments.

In Eukaryotes, both RNA and DNA viruses can also use the EV secretion system to package a huge number of virions (Altan-Bonnet and Chen 2015; Altan-Bonnet 2016). The production of such atypical EVs (hereafter referred to as virions packaging vesicles, VPV) was observed for the first time with polioviruses and rhinoviruses that can accumulate hundreds to thousands of virions in large EVs (Chen *et al.*^2015^). These VPV are produced from autophagosomes i.e. intracellular vesicles with double membranes that originate from the endoplasmic reticulum (Chen *et al.*^2015^). Recently, VPVs carrying huge numbers of virions were also observed during infection of amoeba with *Marseillevirus*, a large DNA virus of the NCLDV superfamily (Fig. 3) (Arantes *et al.*^2016^), suggesting that production of VPVs carrying virions is probably a widespread strategy of eukaryotic viruses. Amoeba infected by *Marseillevirus* produce giant VPVs containing thousands of virions (Fig. 3) that can promote phagocytosis. These VPVs are also apparently formed by the recruitment of membranes from the endoplasmic reticulum and can be surrounded by one of two membranes, resembling the autophagosome. Their release into the environment appears to require cell lysis.

VPVs containing either *Enterovirus* or *Marseillevirus* virions are infectious and can trigger high multiplicity infections after fusion with the host cell membrane (Altan-Bonnet 2016; Arantes *et al.*^2016^). Notably, in both systems, the infection efficiency of VPVs turned out to be much higher than those of free virions (Chen *et al.*^2015^; Arantes *et al.*^2016^). The high multiplicity of infection could enhance the overall fitness of the viral population, facilitating biochemical complementation and genetic recombination, and promoting genetic cooperativity among viral quasi-species (Altan-Bonnet 2016). VPVs are presently unknown in Archaea and Bacteria.

The structural similarity between virions and EVs raises *a priori* practical problems for counting viral particles in environmental samples since one cannot discriminate by electron microscopy between EVs and tailless viruses (Soler *et al.*^2015^). Furthermore, the association of EVs with DNA makes EVs fluorescent after DNA staining, such that EVs and virions can be confused in epifluorescence microscopy, a technique widely used by microbial ecologists (Soler *et al.*^2008^). As a result, it has been suggested that the number of true virions has possibly been overestimated in ecological studies (Forterre *et al.*^2013^; Soler *et al.*^2015^). However, Biller *et al.* (2017) concluded that this might not be the case in marine environments since they only observed a small decrease of epifluorescence-visible EVs upon chloroform treatment from natural marine samples. Chloroform treatment allows discrimination of EVs from tailed viruses (Caudovirales) that do not contain lipids in their virions. Recently, a new method based on interferometry was used to discriminate between EVs and caudovirales virions in environmental samples (Roose-Amsaleg *et al.*^2017^). In this case, they found that tailed virus particles represented between 40 and 70% of all particles present in their river samples, suggesting that the ratio between EVs and viral particles could differ in various environments.

Strikingly, evolutionarily unrelated enveloped viruses infecting eukaryotes have converged in their use of the host machinery for exosome formation to promote their own budding. For example, some non-enveloped RNA viruses (picornavirus) exit from the cell without lysis by hijacking the cellular membrane to cover their capsid, thereby protecting the virion from antibody-mediated neutralization by their host (Feng *et al.*^2013^). Similarly, human immunodeficiency virus, Ebola virus, rabies virus and herpes simplex virus 1 all have well-characterized strategies to hijack members of the ESCRT pathway. These enveloped viruses carry structural Gag proteins that package an RNA genome and present specific viral glycoproteins on their surface, thus enabling them to infect target cells (Votteler and Sundquist 2013). The diversity of EV populations and components suggests that EVs enter cells through various mechanisms similar to the multiple pathways identified for viruses (Marsh and Helenius 2006).

The relationship between mechanisms involved in the production of retroviruses and exosomes and other aspects of their physiology is an active topic of investigation (Izquierdo-Useros *et al.*^2011^; Madison and Okeoma 2015). The formation of intraluminal vesicles within the MVB and the budding of enveloped virions share many features. Both processes require induction of membrane curvature, inclusion of specific cargo and membrane fission for release. Several models have been proposed to explain the similarities found between retroviruses and exosomes: either exosomes are the ancestors of retroviruses or retroviruses merely exploit the same cellular machinery designated for exosome biosynthesis (Gould, Booth and Hildreth 2003; Pelchen-Matthews, Raposo and Marsh 2004). In the latter case, one can even imagine a possible viral origin of the EV system or perhaps an evolutionary conserved system of virus-vesicle co-dependence (Izquierdo-Useros *et al.*^2011^).

A recent study describing eukaryotic-like virus budding in Archaea provides a new interesting model to study the similarities between EVs and virion production (Quemin *et al.*^2016^). The authors reported that the assembly and egress processes of the fusellovirus SV1 infecting *Sulfolobus* are concomitant and occur at the cellular cytoplasmic membrane via a process highly reminiscent of the budding of enveloped viruses that infect eukaryotes. This includes a step in which the lipid containing envelope of the virions fuse or split with the cytoplasmic membrane. Interestingly, this work also revealed the formation of constricted ring-like structures which resemble the budding necks observed prior to ESCRT machinery-mediated membrane scission (Quemin *et al.*^2016^). Since fuselloviruses have also been isolated from Thermococcales (Geslin *et al.*^2003^; Gorlas *et al.*^2012^), they could be useful models to study EV production in these hyperthermophilic archaea.

Considering the similarities between EVs and viral particles, it is tempting to make a link between EVs and the origin of viruses. Two different scenarios involving lipid vesicles have been proposed to explain this origin. Jalasvuori and Bamford (2008) suggested that protocells containing RNA produced vesicles that were used to infect ‘empty’ lipid vesicles, promoting the dissemination of RNA. They called these vesicles ‘protoviruses’. However, defining viruses without referring to capsid proteins is problematic because it makes it impossible to distinguish between viruses and other mobile elements, such as plasmids, transposons and so on (Krupovič and Bamford 2010). If viruses are defined as ‘capsid-encoding organisms’, i.e. producing at least one protein (Raoult and Forterre 2008), they should have only appeared after the emergence of the ribosome. In that framework, Forterre and Krupovič (2012) have suggested that the first viruses originated in the period between formation of the ribosome and LUCA. In that scenario, the emergence of a mechanism for virion formation, excretion and dissemination via transfer is the crucial step in virus origin. Forterre and Krupovic (2012) suggested different mechanisms for virion formation by exaptation of ancient cellular systems, explaining the different types of virions present in modern viruses. Among them, the production of EVs could have been at the origin of modern viruses, such as retroviruses, plasmaviruses or pleolipoviruses.

### EVs in the history of life

The production of EVs by cells from the three domains of life has suggested that the LUCA and its contemporaries already produced EVs (Gill and Forterre 2016). Comparative genome analysis indeed suggests that LUCA was already a rather sophisticated cellular organism that already harbored a cytoplasmic membrane (Pereto, Lopez-Garcia and Moreira 2004; Jekely 2006; Koonin 2006; Forterre and Gribaldo 2007). Proteins whose origin can be traced back to LUCA include several subunits of the membrane-bound ATP synthase, signal recognition particles involved in the translation of membrane proteins and Sec proteins involved in protein secretion. Phylogenomic analyses also predict that LUCA had unsaturated polyisoprenols (Lombard 2016). Since these universal molecules are used today as lipid carriers in glycosylation pathways, this suggests that LUCA possibly had some form of glycoprotein layer covering the cytoplasmic membrane. However, the fact that LUCA had a membrane does not necessarily mean that it was able to produce EVs. To clarify this point, it would be important to determine if homologous machineries for EV formation exist in Archaea and Bacteria and can be traced back to LUCA. We are thus limited in our conclusion since the mechanisms of EV production in the three domains remain poorly understood, especially in Archaea. Among proteins usually found in EVs, the only possible universal proteins could be proteins of the SPFH superfamily that are known to facilitate membrane curvature and cell fusion (Lee *et al.*^2017^) and have been involved in the production of some eukaryotic EVs (Hinderhofer *et al.*^2009^). However, these are small proteins with multiple paralogs, especially in eukaryotes, and their presence in LUCA is difficult to ascertain (Browman, Hoegg and Robbins 2007; Hinderhofer *et al.*^2009^). It would be thus important to get more insight on the possible role of these proteins in EV production and to update their phylogenomic analysis. Finally, the discovery that overexpression of hyaluronan synthase induces the formation of nanopods and the production of microvesicles in eukaryotes (Rilla *et al.*^2013^; Koistinen *et al.*^2017^) is possibly interesting because homology of hyaluronan synthase is present in Bacteria and Archaea. However, it is not known if hyaluronan is a component of EVs in these two domains. It would be interesting to know if overexpression of hyaluronan synthase also stimulates EV production in Bacteria and if homologs of this enzyme in Archaea have the same activity.

Different and possibly redundant mechanisms for EV production probably originated after LUCA, during the divergence of the three domains. The emergence of peptidoglycan in Bacteria probably led to a drastic modification in EV structure with the appearance of OMV (depleted in cytoplasmic components) and O-IMV. The emergence of the ESCRT system in the lineage common to Archaea and Eukarya suggests that this mechanism was used for EV production in the last common ancestor of both domains. However, the loss of the ESCRT system in some Archaea (e.g. Thermococcales) that produce abundant vesicles clearly indicates that several redundant systems may be involved in EV production.

Recently, two original scenarios were proposed that accord an important role for EV production in the origin of Eukaryotes. Baum and Baum (2014) suggested that the eukaryotic cytoplasm originated from the expansion of EVs and nanopods produced by an archaeon. These EVs and nanopods of increasing sizes entrapped the bacterium at the origin of mitochondria and fused to produce both a continuous cytoplasmic membrane and the endoplasmic reticulum with its extensions e.g. the nuclear membrane or the Golgi apparatus. However, it is unclear in this model how the archaeal lipids of the EVs/nanopods were transformed into the ‘bacterial-like’ lipids of the modern eukaryotic endomembrane system. Furthermore, interactions between archaeal nanopods and bacteria have not yet been observed in nature.

In a more classical model, Martin and co-workers suggested that the eukaryotic intracellular membrane system originated from OMVs produced by the bacterial ancestors of mitochondria inside the cytoplasm of an archaeal host (Gould, Garg and Martin 2016). The lipids from these OMVs progressively replaced all previous archaeal lipids of the host, including the cytoplasmic membrane. This model is based on the observation that modern mitochondria still produce EVs that can transfer materials between the mitochondrion and eukaryotic intracellular vesicles (peroxysomes, lysosomes, etc.) (Andrade-Navarro, Sanchez-Pulido and McBride 2009; Soubannier *et al.*2012a). Similar to OMVs produced by free-living bacteria, mitochondrial EVs are overexpressed under stress conditions and seem to be involved in the detoxification of damaged compounds such as oxidized proteins (Soubannier *et al.*2012b).

Although the above scenarios are somehow appealing, one should bear in mind that we have presently no examples of an archaeon engulfing a bacterium with its nanopods or of an intracellular bacteria thriving in an archaeon, as postulated by the above scenarios. The hypothesis that an archaeon was transformed into a eukaryote during eukaryogenesis was recently boosted by the discovery of the putative Asgard superphylum and phylogenetic analyses supporting their sisterhood with eukaryotes (Spang *et al.*^2015^; Zaremba-Niedzwiedzka *et al.*^2017^). However, these phylogenetic analyses have been questioned due to the inclusion of many fast-evolving species in the species dataset used for these analyses (Da Cunha *et al.*^2017^; for controversies surrounding these data, see Da Cunha *et al.*^2018^; Spang *et al.*^2018^). Universal trees of life based on the longer universal proteins, especially RNA polymerase large subunits, strongly support the classical Woese tree of life (Da Cunha *et al.*^2017^). If the bacterial ancestor of mitochondria did not invade an archaeon but a proto-eukaryote, EVs produced by ancient bacteria living as endosymbionts in proto-eukaryotes (including the mitochondrial ancestor) could still have played an important role in eukaryogenesis by interacting with the intracellular EVs network of proto-eukaryotes.

## CONCLUDING REMARKS

Research on EVs from cells of all three domains of life has seen an explosion of interest in the last decade. Increasing evidence indicates that these EVs impact intra- and intercellular communication by the transfer of a myriad of biomolecules and the exchange of genetic information. Despite the increased research interest, the accumulating data on EVs are increasingly complex, leaving many fundamental questions unanswered, such as the mechanisms of cargo sorting and biogenesis. The discovery of nanotubes associated with EVs in all three domains of life is highly intriguing, suggesting that the formation of tubular structures is a universal mechanism for cell-to-cell communication and for the transport of EVs between cells.

In Bacteria, vesiculation appears to be a specific and regulated process. Given the diversity and complexity of different bacterial envelopes, it is not surprising that the mechanisms involved in biogenesis are not yet fully understood. Indeed, it may be that there is no single mechanism for all bacteria. Nonetheless, understanding how diverse bacteria achieve a common outcome will benefit both the bacterial and EV fields in general. Diverse factors such as iron and oxygen availability; biofilm versus planktonic lifestyle; SOS response; exposure to antibiotics; host factors; and growth conditions affect the composition and quantity of EVs (reviewed in Orench-Rivera and Kuehn 2016). Future studies addressing how EVs are influenced by different environmental conditions will hopefully advance our current understanding.

A major problem in the eukaryotic field is the isolation and purification of different subsets of EVs from complex mixtures. Protocols currently used for purifying EVs usually result in isolating a mixture of different EVs with various origins (microvesicles, exosomes, etc.) along with other macromolecular complexes. Moreover, as different subsets of EVs contain many common markers, this poses a problem for EV typing. In order to standardize research between different laboratories, it has been suggested that future studies should try and analyze a minimum of three or more proteins *expected* to be present in the EVs studied using approaches such as western blots, flow cytometry (FACS) or proteomic analysis (Lötvall *et al.*^2014^). More recently, a knowledge base termed EV-TRACK was set up to encourage increased systematic reporting of EV biology and methodology (van Deun *et al.*^2017^). Such additional measures would reinforce conclusions regarding the functions of EVs and their roles.

Though slow to start, research on archaeal EVs is showing promising results in linking mechanisms of vesicle production between domains of life. The presence of eukaryotic-like mechanisms (e.g. ESCRT) in prokaryotic organisms is a major asset in identifying a unified mechanism of EV production. However, even in Archaea, it has become apparent that many mechanisms may be required to explain the diversity of EVs observed. A major future challenge in the field of EVs will certainly be to identify specific mechanisms involved in membrane fusion and vesicle biogenesis in Archaea with monolayer membranes, since models proposed up to now are based on the ‘classical’ bacterial and eukaryotic bilayer membranes. Regardless of the mechanism, it is clear that the local structure and organization of lipid membranes plays an important role in EV biogenesis across all three domains of life.

The connection between viruses and EVs also reveals important connections in EV production and function across the three domains of life. Whether viruses have hijacked EV machinery to their own benefit, or whether EVs sit at the origin of modern viruses, it is clear that these entities are intimately linked. There appears to exist a continuum between pure vesicles and pure virions, interspersed with vesicles containing nucleic acids, plasmidions, membrane-encapsulated viruses, viral vesicles, VPV, etc. No doubt future research into both viruses and EVs will provide mutual insights to help illuminate this fascinating relationship.
